# Supplementary material for: Dietary Uncoupling of Gut Microbiota and Energy Harvesting from Obesity and Glucose Tolerance in Mice
Source: Cell Rep. 2017 Nov 7;21(6):1521–33. doi: 10.1016/j.celrep.2017.10.056 (PMC5695904; doi:10.1016/j.celrep.2017.10.056)
Supplement: Document S1. Figures S1–S4 [file mmc1.pdf]

Cell Reports, Volume *21*

Supplemental Information

Dietary Uncoupling of Gut Microbiota and Energy Harvesting from Obesity and Glucose Tolerance in Mice

Matthew J. Dalby, Alexander W. Ross, Alan W. Walker, and Peter J. Morgan

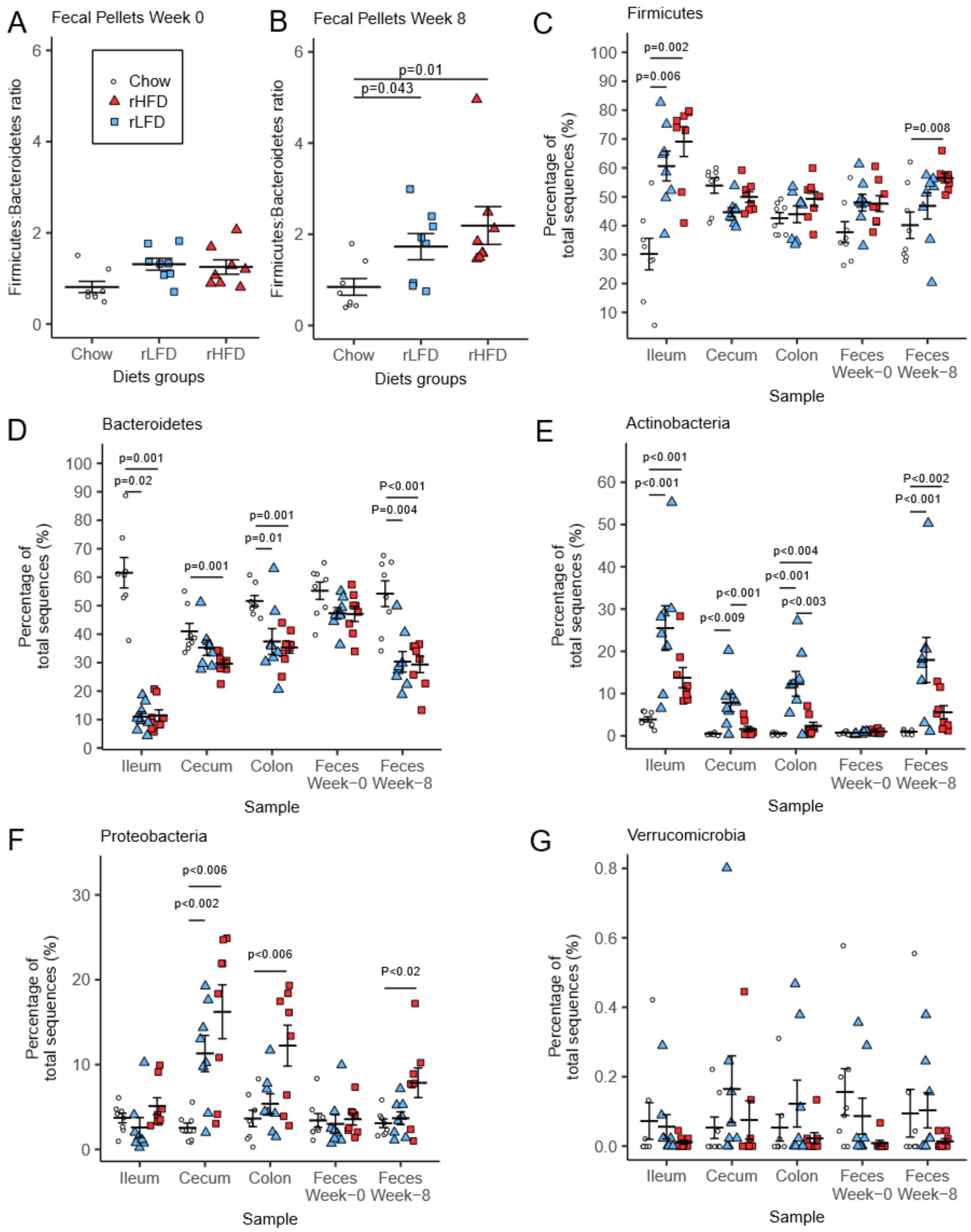

Figure S1. Initial F:B ratio and phylum proportional abundance. Related to Figure 1.

(A) Firmicutes:Bacteroidetes (F:B) ratio at Week 0. (B) F:B ratio at Week 8. Proportional abundance of the (C) Firmicutes phylum, (D) the Bacteroidetes phylum, (E) the Actinobacteria phylum, (F) the Proteobacteria phylum, and (G) the Verrucomicrobia phylum. Data represent mean ± SEM. n = 8 mice/group.

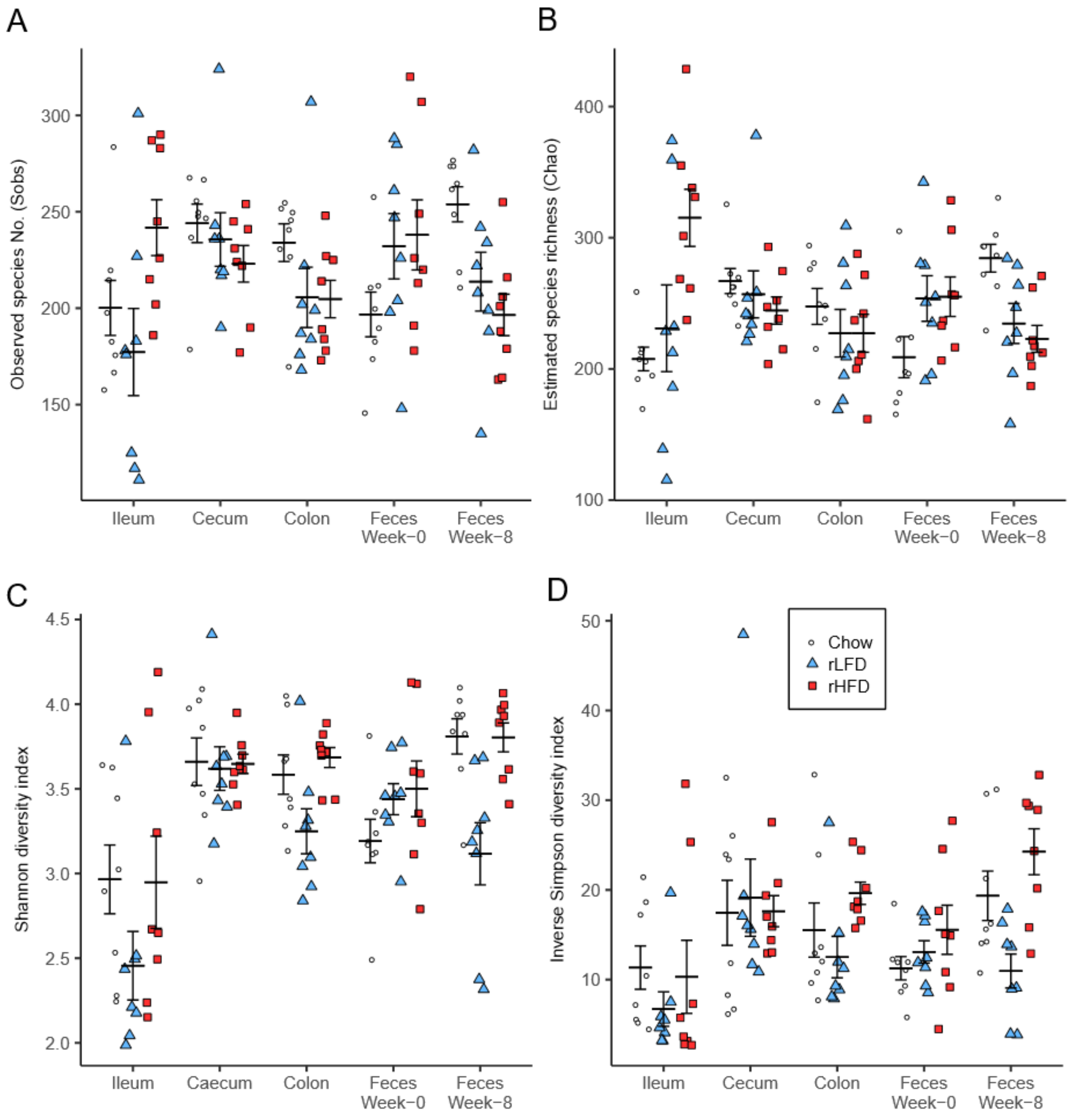

Figure S2. Measures of microbiota diversity. Related to Figure 1.

(A) Observed species richness (Sobs). (B) Estimated total species richness (Chao). (C) Shannon diversity index. (D) Inverse Simpson diversity index. Data represent mean ± SEM. n = 8 mice/group.

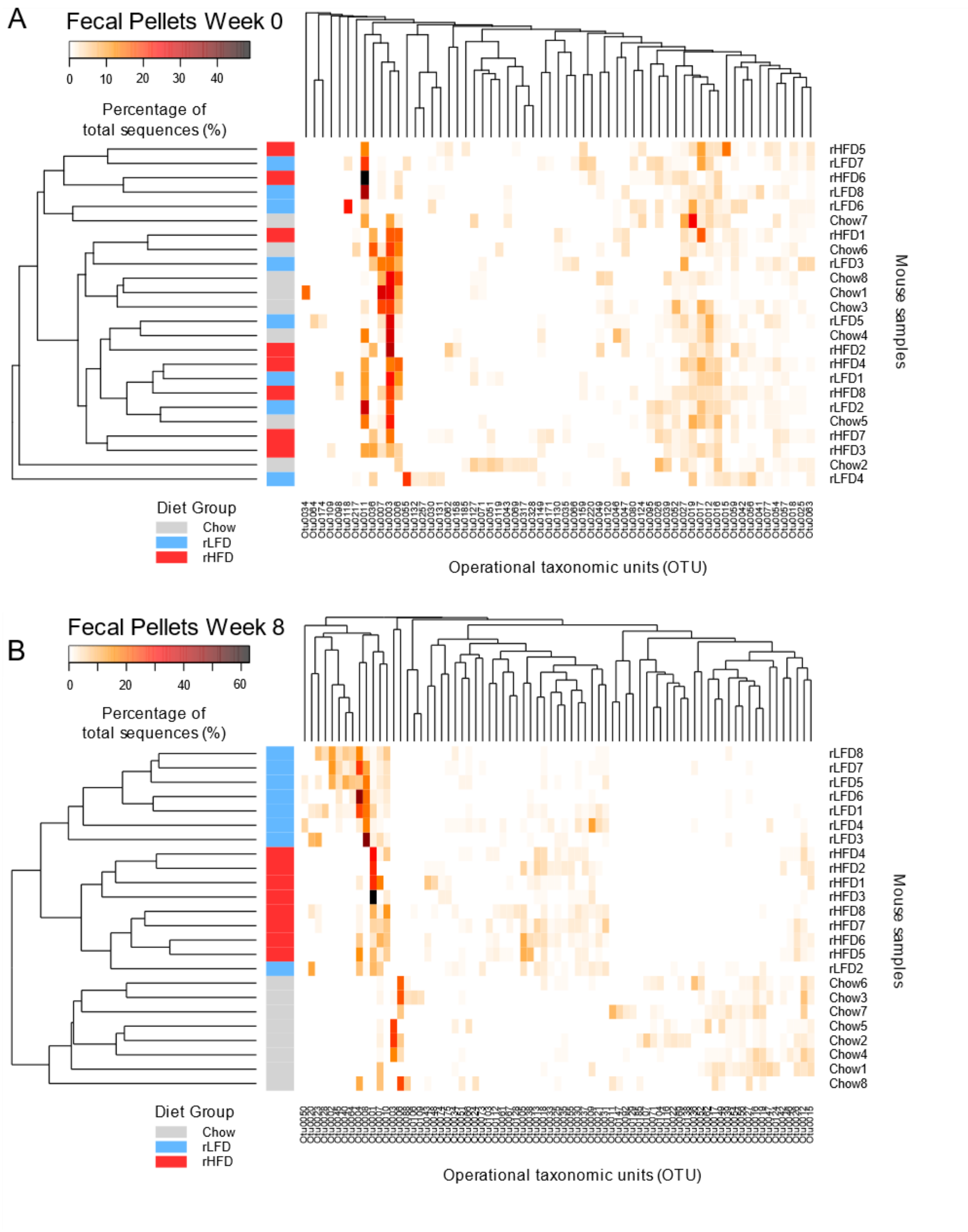

Figure S3. Effect of diet on fecal pellet microbiota composition. Related to Figure 3.

(A) Fecal pellet microbiota composition at Week 0. (B) Fecal pellet microbiota composition at Week 8. Heat maps show proportions of OTUs (>3% abundance) in the fecal pellets, with rows clustered by microbiota similarity using the Bray-Curtis calculator, and columns clustered by OTUs that occur more often together in a sample. n = 8 mice/group.

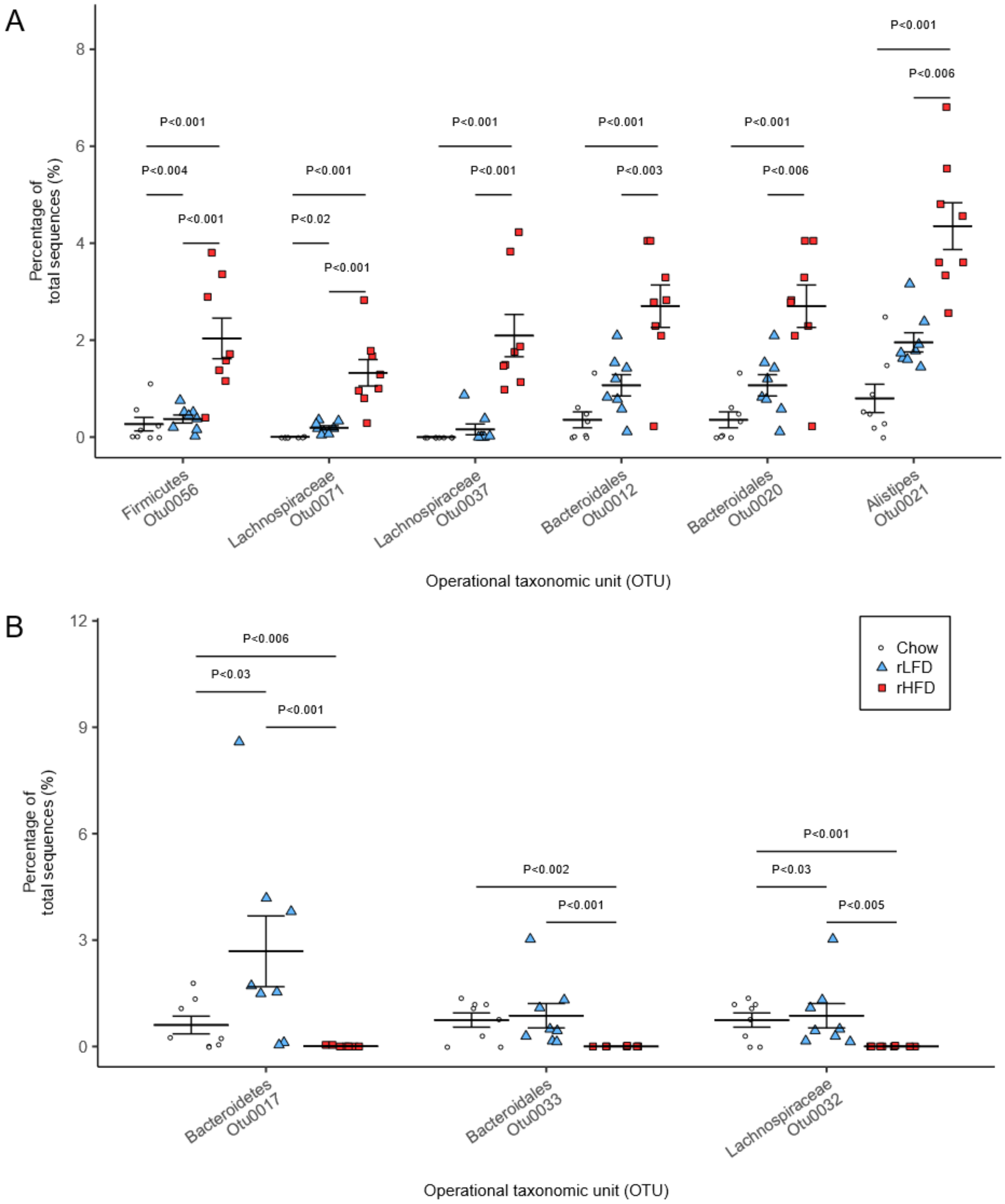

Figure S4. Cecal OTUs with abundance significantly altered only in rHFD fed mice. Related to Figure 4.

(A) OTUs (>0.5% proportional abundance) with significantly higher proportional abundance in rHFD fed mice compared to both Chow and rLFD fed mice. (B) OTUs (>0.5% proportional abundance) with significantly lower proportional abundance in rHFD fed mice compared to both Chow and rLFD fed mice. Statistical significance determined using Metastats in the mothur program. Data represent mean ± SEM. n = 8 mice/group.
