# Supplementary material for: Dietary Uncoupling of Gut Microbiota and Energy Harvesting from Obesity and Glucose Tolerance in Mice
Source: Cell Rep. 2017 Nov 7;21(6):1521–33. doi: 10.1016/j.celrep.2017.10.056 (PMC5695904; doi:10.1016/j.celrep.2017.10.056)
Supplement: Document S2. Article plus Supplemental Information [file mmc4.pdf]

Cell Reports

Article

Dietary Uncoupling of Gut Microbiota and Energy Harvesting from Obesity and Glucose Tolerance in Mice

Graphical Abstract

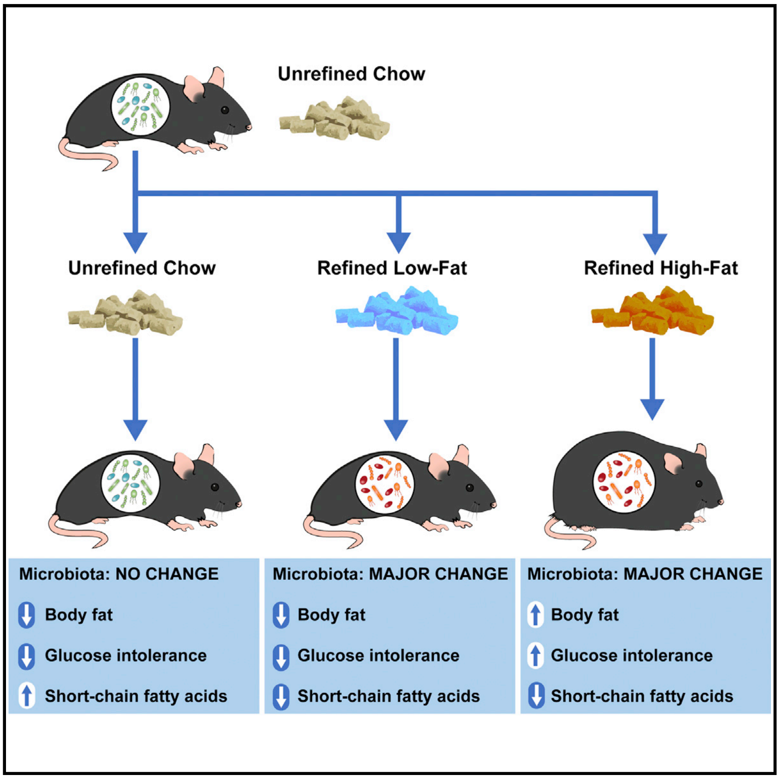

Highlights

- High-fat diet changes in mouse gut microbiota composition due to diet, not obesity
- Glucose intolerance in mice linked to high-fat diet, not changes in gut microbiota
- Cecal fermentation (energy harvest) decreased, not increased, by high-fat diet
- Choice of control diet is vitally important to studies of microbiota composition

Authors

Matthew J. Dalby, Alexander W. Ross, Alan W. Walker, Peter J. Morgan

Correspondence

p.morgan@abdn.ac.uk

In Brief

Dalby et al. show that high-fat-diet-induced changes in gut microbiota composition and cecal fermentation can be dissociated from obesity and glucose intolerance in mice by using a nutritionally more appropriate control diet. This highlights the importance of the control diet in interpreting microbiota studies.

Dalby et al., 2017, Cell Reports *21*, 1521–1533
November 7, 2017 © 2017 The Authors.
https://doi.org/10.1016/j.celrep.2017.10.056

OPEN ACCESS
CellPress

Dietary Uncoupling of Gut Microbiota and Energy Harvesting from Obesity and Glucose Tolerance in Mice

Matthew J. Dalby,[1] Alexander W. Ross,[1] Alan W. Walker,[1] and Peter J. Morgan[1,2,*]
[1]Rowett Institute, University of Aberdeen, Foresterhill, Aberdeen AB25 2ZD, UK
[2]Lead Contact
*Correspondence: p.morgan@abdn.ac.uk
https://doi.org/10.1016/j.celrep.2017.10.056

SUMMARY

Evidence suggests that altered gut microbiota composition may be involved in the development of obesity. Studies using mice made obese with refined high-fat diets have supported this; however, these have commonly used chow as a control diet, introducing confounding factors from differences in dietary composition that have a key role in shaping microbiota composition. We compared the effects of feeding a refined high-fat diet with those of feeding either a refined low-fat diet or a chow diet on gut microbiota composition and host physiology. Feeding both refined low- or high-fat diets resulted in large alterations in the gut microbiota composition, intestinal fermentation, and gut morphology, compared to a chow diet. However, body weight, body fat, and glucose intolerance only increased in mice fed the refined high-fat diet. The choice of control diet can dissociate broad changes in microbiota composition from obesity, raising questions about the previously proposed relationship between gut microbiota and obesity.

INTRODUCTION

The gut microbiota is a key interface for nutrition, with dietary substrates able to shape microbial composition, having important metabolic consequences for the host (Turnbaugh et al., 2006, 2008). Germane to this is an "obese microbiota," with a composition characteristic of the obese state, that is also able to induce obesity (Ley et al., 2005; Turnbaugh et al., 2006, 2008). This is supported by studies in which the transfer of fecal microbiota from obese donors (murine or human) to recipient germ-free mice results in increased body fat (Ridaura et al., 2013; Turnbaugh et al., 2008). Additionally, obesogenic high-fat diets reportedly result in changes in gut microbial composition in mouse models (Turnbaugh et al., 2008). Proposed characteristics of an obese microbiota include an increased Firmicutes-to-Bacteroidetes (F:B) ratio (the predominant bacterial phyla in the human and mouse gut), reductions in microbial diversity, and changes in specific bacterial families, or species (Ley et al., 2006; Turnbaugh et al., 2009).

While several mechanisms have been proposed by which an "obese microbiota" can influence body weight homeostasis (Bäckhed et al., 2004; Byrne et al., 2015; Cani et al., 2007; Stenman et al., 2012), increased energy harvest via colonic fermentation and short-chain fatty acid (SCFA) production is the most direct (Turnbaugh et al., 2006). Nonetheless, the evidence for energy harvest was based on transplantation studies of microbiota from genetically obese (*ob/ob*) mice into germ-free wild-type recipients (Turnbaugh et al., 2006). What is not yet clear is whether an obese microbiota, with increased capacity for energy harvest, results from feeding a high-fat diet to normal mice.

High-fat diets, containing a large percentage of energy from fat, have become a standard model for inducing obesity in mice (Buettner et al., 2007; Black et al., 1998; West et al., 1995). Studies investigating the role of gut microbiota in diet-induced obesity have frequently compared mice fed a refined high-fat diet (rHFD), in which each nutrient is supplied by a specific and purified ingredient, to mice fed a chow diet (Cani et al., 2007; Everard et al., 2013; Turnbaugh et al., 2006; Warden and Fisler, 2008). While commonly referred to as "standard chow," there is no standardization among the chow diets used, and their nutritional composition differs significantly from those of rHFDs (Warden and Fisler, 2008). Chow diets typically contain a large proportion of dietary fiber from unrefined cereals and legumes that is absent from rHFDs (Ricci, 2013).

Given these differences, a refined low-fat diet (rLFD), formulated using the same purified ingredients as the rHFD used, is a more appropriate control diet than chow (Ricci, 2013). Recent research suggests that mice fed an rLFD have increased adiposity relative to chow-fed mice, indicating that these diets are not nutritionally equivalent (Benoit et al., 2013; Chassaing et al., 2015). The lack of fermentable fiber in the refined diet was a potential explanation for this increased adiposity (Chassaing et al., 2015). As fiber is one of the main dietary substrates shaping gut microbiota composition (David et al., 2014), these results indicate that diet composition is a key consideration when examining the relationship between gut microbes and obesity. The use of chow diets with inappropriately matched nutritional composition has the potential to produce misleading results, and the impact of this as a confounding factor on microbiota composition changes in diet-induced obese mice has not previously been investigated.

In this study, we examine the effect of an rHFD in C57BL/6J mice on gut microbiota composition, adiposity, glucose control,

This is an open access article under the CC BY license (http://creativecommons.org/licenses/by/4.0/).

OPEN ACCESS
CellPress

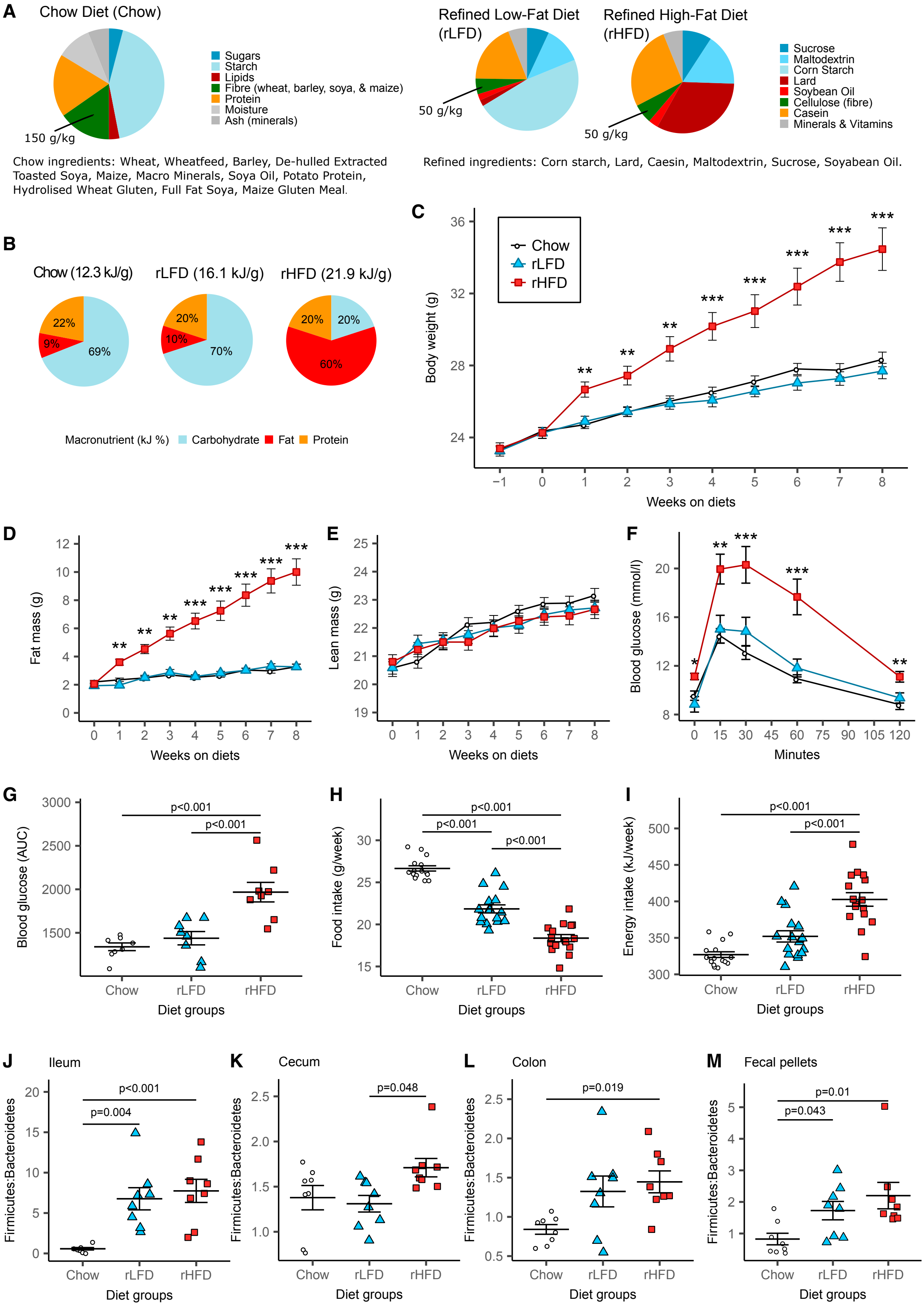

(legend on next page)

and capacity for energy harvest from SCFAs relative to mice fed two control diets, a typical chow diet, or an rLFD with a nutritional composition matched to the rHFD. The results show that the choice of control diet profoundly influences the outcome of the study and its interpretation.

RESULTS

Dietary Fat Drives Obesity and Glucose Intolerance Independently of Refined Diet Ingredients

To investigate the contribution of dietary fat and nutritional composition to obesity, mice were fed either an rHFD, a matched rLFD, or a chow diet (Chow) for 8 weeks. The chow diet was formulated from relatively unrefined ingredients, while the refined diets were both formulated using the same nutritionally defined and purified ingredients (Figure 1A). The rHFD and rLFD contained 5% dietary fiber as cellulose, while the chow diet contained 15% dietary fiber as complex plant polysaccharides. Both the chow diet and rLFD contained a similar macronutrient ratio (Figure 1B).

The body weights and body fat of mice fed chow diet or an rLFD did not differ during the study (Figures 1C and 1D). In contrast, rHFD-fed mice rapidly increased in body weight and body fat, gaining more than chow diet- or rLFD-fed mice (Figures 1C and 1D). The lean mass of mice fed an rHFD, rLFD, or chow diet remained the same during the study (Figure 1E). As lean mass remained unchanged, the increasing body weight in rHFD-fed mice was due to increased body fat.

After 8 weeks, fasting blood glucose was higher, and blood glucose was elevated following intraperitoneal glucose injection in rHFD-fed mice relative to chow diet- and rLFD-fed mice (Figure 1F). The area under the curve for blood glucose was higher in rHFD-fed mice relative to both chow diet- and rLFD-fed mice (Figure 1G). There was no difference in blood glucose measures between rLFD- and chow diet-fed mice (Figures 1F and 1G). Food intake was reduced in rLFD-fed mice compared to chow diet-fed mice, and it was further reduced in rHFD-fed mice relative to both rLFD- and chow diet-fed mice (Figure 1H). Energy intake was increased in the rHFD-fed mice compared to both the chow diet- and rLFD-fed mice, but it did not differ between the chow diet- and rLFD-fed mice (Figure 1I), due to the differences in dietary energy density (Figure 1B).

Refined Diets Increase the Firmicutes-to-Bacteroidetes Ratio and Reduce Diversity Independently of Obesity or Dietary Fat

To investigate the effects of diet composition on the intestinal microbiota, we sequenced the V1–V2 variable region of the 16S rRNA gene initially in the fecal pellets when all mice were fed chow diet and after 8 weeks of feeding the experimental diets in the luminal contents of the ileum, cecum, and colon and in the fecal pellets. Bacterial OTUs (based on V1–V2 16S rRNA gene sequences clustered at 97% sequence similarity) were calculated as a proxy for bacterial ‘‘species.’’ Initial analysis identified an OTU corresponding to *Lactococcus lactis* in rLFD- and rHFD-fed mice becoming the dominant OTU in the ileum of these mice but not in chow diet-fed mice (Table S1). This *Lactococcus lactis* sequence has been identified as probable contaminating DNA contained in the refined diets used (Dollive et al., 2013). The 16S rRNA gene sequence data were, therefore, processed to remove sequences corresponding to the genus *Lactococcus*, and these cleansed data were then used in all subsequent analyses (Table S2).

The Firmicutes:Bacteroidetes (F:B) ratio increased in the ileum, colon, and fecal pellets of mice fed both the rHFD and rLFD relative to chow diet-fed mice (Figures 1J, 1L, and 1M). The F:B ratio in fecal pellets was initially similar in all mice (Figure S1A). The F:B ratio was unaffected by diet in the cecum (Figure 1K). The microbiota was dominated by the Firmicutes and Bacteroidetes phyla (Figures S1C and S1D), with lower proportions of Actinobacteria, Proteobacteria, and Verrucomicrobia (Figures S1E–S1G).

Next, we examined the effect of diet on microbial diversity. Observed species richness (Sobs), estimated species richness (Chao), the Shannon diversity index, and the inverse Simpson diversity index were unchanged between diet groups (Figures S2A–S2D). Initial microbial diversity in fecal pellets at week 0 was similar across all mice (Figure S2).

Refined Diets Drive Extensive Alterations in Ileum and Cecum Microbiota Composition

Mice fed an rLFD or rHFD showed extensive alterations in the composition of the gut microbiota compared to the chow diet-fed mice. The overall similarity of the ileum microbiota between each mouse, calculated using a Bray-Curtis matrix, shows that ileal samples from mice fed both an rLFD and an rHFD clustered together but were distinct from chow diet-fed mice (Figure 2A).

Figure 1. Effects of Chow Diet, rLFD, and rHFD on Mouse Physiology and Phylum Ratio
(A) Composition of diet nutritional composition and ingredients.
(B) Macronutrient proportions of the diets.
(C) Weekly body weight.
(D) Weekly body fat.
(E) Weekly lean mass.
(F) Blood glucose concentrations following intraperitoneal glucose tolerance tests.
(G) Blood glucose area under the curve (AUC).
(H) Mean weekly food intake.
(I) Mean weekly energy intake.
(J–M) Firmicutes-to-Bacteroidetes (F:B) ratio in (J) the ileum, (K) the cecum, (L) the colon, and (M) fecal pellets.
Data indicate mean ± SEM. $*p < 0.05$, $**p < 0.01$, $***p < 0.001$. n = 16 mice/group for (C), (D), (E), (H), and (I). n = 8 mice per group for (F), (G), (J), (K), (L), and (M).
See also Figures S1 and S2 and Table S2.

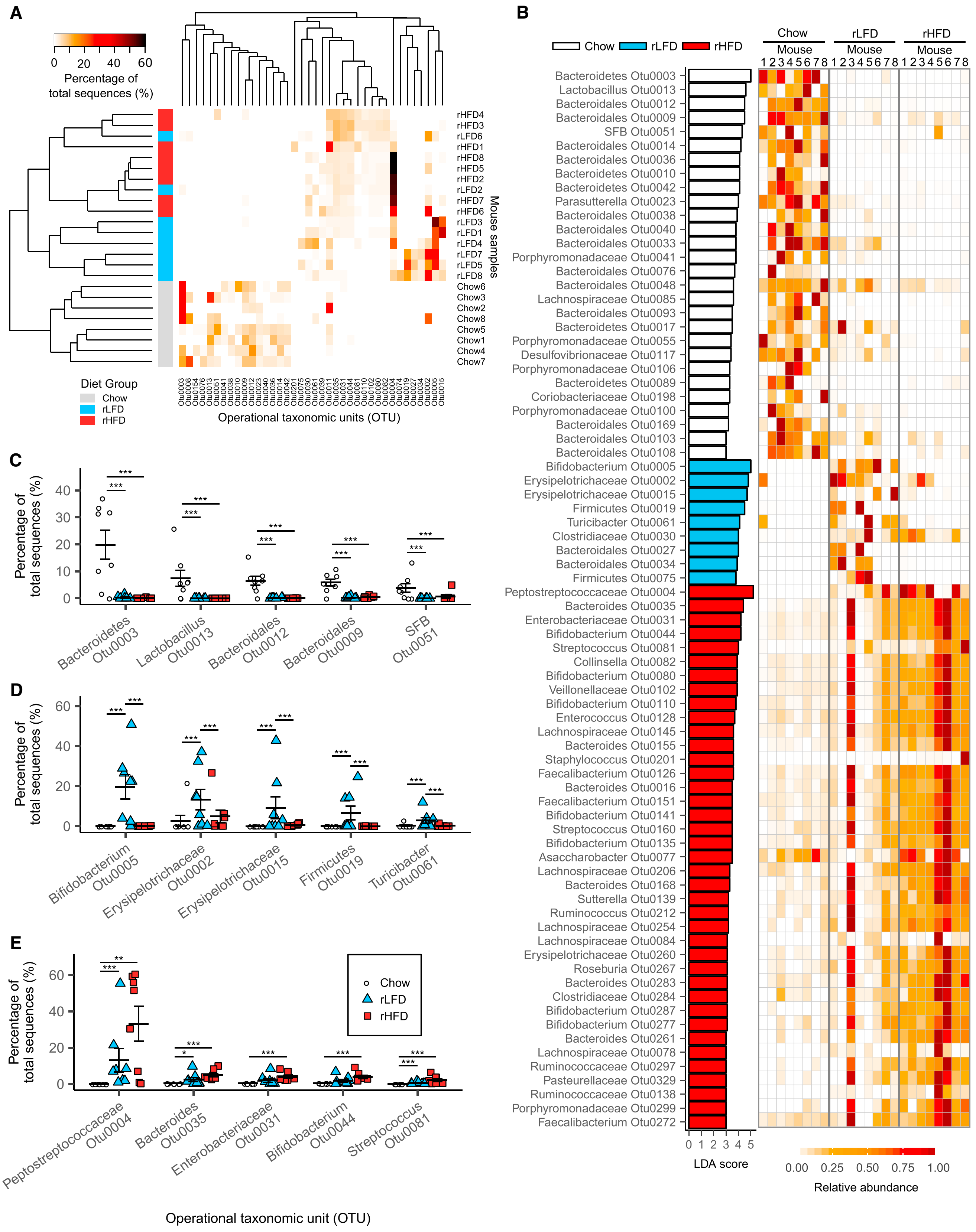

(legend on next page)

The heatmap shows that the proportionally most abundant OTUs in the ileum microbiota in chow diet-fed mice were replaced in rLFD- and rHFD-fed mice by a new and distinct set of OTUs (Figure 2A). The LEfSe program was used to identify OTUs that differed in proportional abundance between the 3 diet treatments, using linear discriminant analysis (LDA) scores (Segata et al., 2011). The bar graph shows the LDA score for ileum OTUs with an LDA score of ≥ 3, while the heatmap shows the relative abundance of each OTU across the ileum samples from each mouse (Figure 2B). The proportional abundance of the 5 OTUs with the highest LDA scores in the chow diet-fed mice was reduced in both rLFD- and rHFD-fed mice (Figure 2C). The proportional abundance of the 5 OTUs with the highest LDA scores in rLFD-fed mice was higher than in both chow diet- and rHFD-fed mice (Figure 2D). In contrast, the 5 OTUs with the highest LDA scores in rHFD-fed mice were also significantly proportionally increased in rLFD-fed mice relative to chow diet-fed mice (Figure 2E).

In the cecum, mice fed the rLFD or rHFD showed similarly extensive changes in the microbiota composition relative to chow diet-fed mice. Using the Bray-Curtis calculator, the microbiota from chow diet-fed mice clustered distinctly from both rLFD- and rHFD-fed mice (Figure 3A). The heatmap shows that the proportionally most abundant OTUs in the cecum microbiota of chow diet-fed mice were largely replaced by different OTUs in rLFD- and rHFD-fed mice (Figure 3A). Similarly, based on individual OTUs with the highest LDA scores, the most characteristic microbiota in the cecum of chow diet-fed mice were displaced in both rLFD-fed and rHFD-fed mice (Figure 3B). As in the ileum, the most highly represented OTUs in the chow diet-fed mice were reduced in both rLFD- and rHFD-fed mice (Figure 3C). For rHFD-fed mice, the most representative OTUs also increased in rLFD-fed mice relative to chow diet-fed mice (Figure 3E). The OTUs with the highest LDA score in the rLFD-fed mice increased relative to both chow diet- and rHFD-fed mice (Figure 3D).

To further explore any changes in microbiota with diet and obesity, the comparisons of microbiota OTU composition were analyzed as paired diets (Figure 4). Comparisons restricted to the chow diet- and rHFD-fed mice only for the ileum and cecum emphasized the distinct microbiota present between the two diet groups (Figures 4A and 4B). A similarly distinct microbiota OTU composition was evident when the chow diet and rLFD groups were compared for the ileum and cecum (Figures 4C and 4D). In contrast, for the rLFD- and rHFD-fed mice, there was no clear separation between the two diet groups in the ileum or cecum (Figures 4E and 4F). Metastats results were used to identify OTUs with an average of $\geq 0.5\%$ of the microbiota in either ileum or cecum that were significantly higher or lower in rHFD-fed obese mice relative to both chow diet- and rLFD-fed lean mice. In total, 6 OTUs increased (Figure S4A), and three OTUs decreased (Figure S4B) only in rHFD-fed obese mice. This included 2 OTUs classified as *Lachnospiraceae* (OTU37 and OTU71) and 2 classified as *Bacteroidales* (OTU12 and OTU20) that increased in proportional abundance in rHFD-fed obese mice, while 1 *Lachnospiraceae* (OTU32) and 1 *Bacteroidales* (OTU33) decreased in rHFD-fed obese mice, compared to both chow diet- and rLFD-fed lean mice (Figure S4). In addition, an OTU classified as an *Alistipes* (OTU21) and an unclassified Firmicute (OTU56) increased only in rHFD-fed mice (Figure S4). Changes in composition that were comparable to those observed in the cecum were also seen in the fecal pellet microbiota (Figure S3B). The initial fecal pellet microbiota composition was similar in all mice fed chow diet at the start of the experiment (Figure S3A).

Refined Diet Low in Fiber Alters Cecal Short-Chain Fatty Acids and Gut Morphology

Concentration and total amounts of acetic, propionic, and butyric acid were reduced in the ceca of rLFD- and rHFD-fed mice compared to chow diet-fed mice (Figures 5A and 5B). Isovaleric acid concentrations were at undetectable levels in chow diet-fed mice compared to those in mice fed an rLFD or rHFD, whereas concentrations of isobutyric acid and valeric acid were unchanged (Figure 5C). Total isobutyric acid was higher in the cecum of chow diet-fed mice, while valeric acid was unchanged (Figure 5D). Mean ratios of acetate:propionate:butyrate were 66:12:22 for chow diet-fed mice, 74:17:9 for rLFD-fed mice, and 78:12:9 for rHFD fed mice, with butyric acid forming a greater proportion of the total SCFAs in chow diet-fed mice.

In rLFD- or rHFD-fed mice, the cecum and colon were visibly smaller and shorter than in mice fed a chow diet (Figure 5E). Compared to mice fed chow diet, intestinal content weights were reduced in the ceca of rLFD- or rHFD-fed mice (Figure 5G), remaining unchanged in the small intestine or colon (Figures 5F and 5H). The tissue weight and length of the small intestine (Figures 5I and 5L), cecum (Figures 5J and 5M), and colon (Figures 5K and 5N) tissue were reduced in rLFD- and rHFD-fed mice compared to mice fed chow diet.

DISCUSSION

Here, we show that body weight, body fat, energy intake, and blood glucose responses did not differ between mice fed rLFD or chow diet. In contrast, increased body weight and adiposity has been previously reported in mice fed an rLFD relative to chow diet (Chassaing et al., 2015). As chow diets are not standardized, the larger proportions of protein in the chow diet and

Figure 2. Effect of Chow Diet, rLFD, and rHFD on Ileum Microbiota Composition

(A) Heatmap of OTUs ($\geq$3% abundance) in the ileum, with rows clustered by microbiota similarity using the Bray-Curtis calculator, and columns clustered by OTUs that occur more often together.

(B) OTU LDA values for chow diet-, rLFD-, and rHFD-fed mice (LDA score > 3) and heatmap showing relative abundance of each OTU between mouse samples across rows; columns represent OTUs within each sample.

(C–E) Dot plots represent proportional abundance of the 5 OTUs with the highest LDA score within (C) the chow diet group, (D) the rLFD group, and (E) the rHFD group; symbols each represent individual mice with mean ± SEM.

Significance determined using Metastats in mothur. *p < 0.05; **p < 0.01; ***p < 0.001. n = 8 mice per group.

See also Table S2.

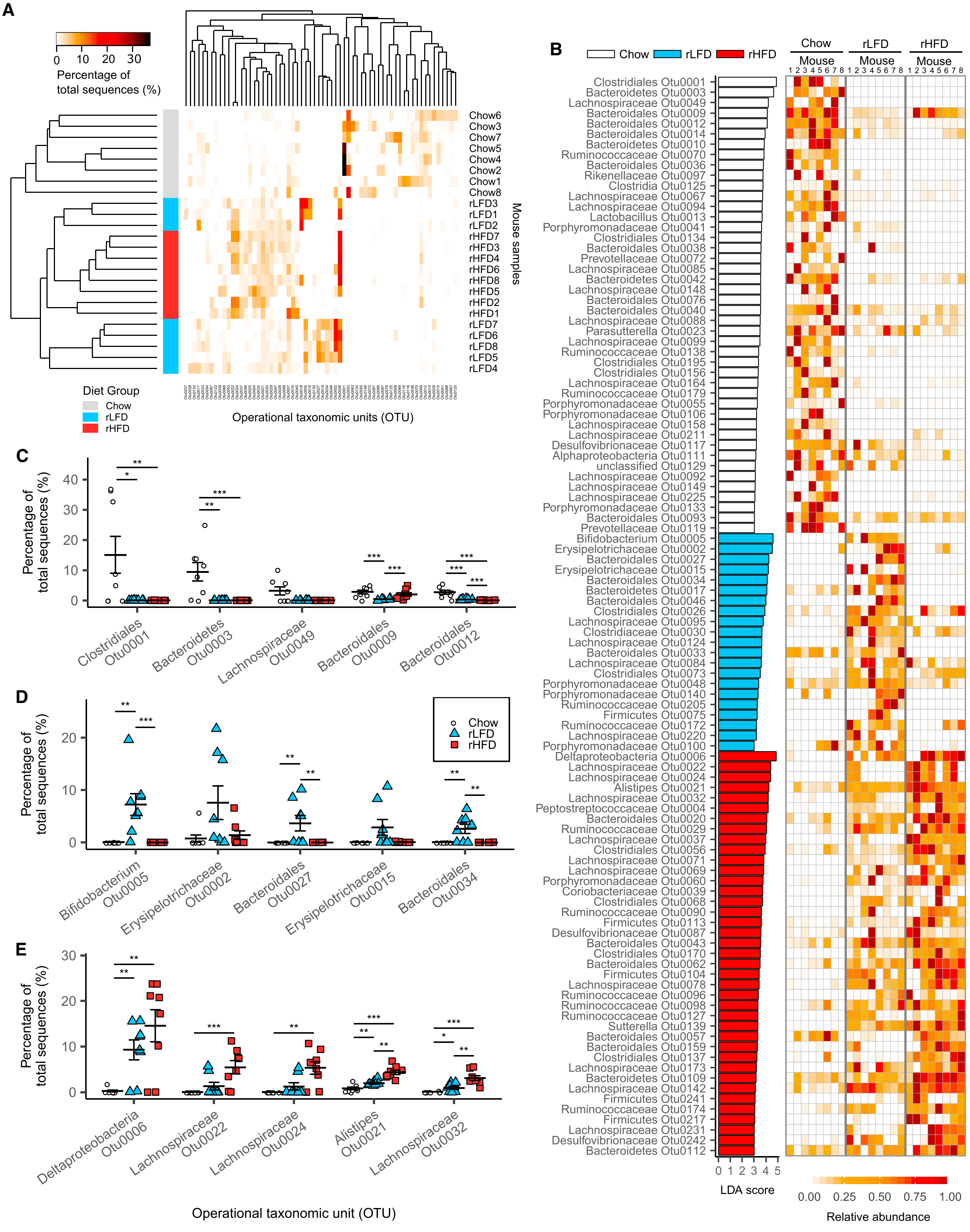

(legend on next page)

sucrose in the rLFD used potentially explain this discrepancy. The chow diet and rLFD used in the present study contained similar proportions of energy from protein and sucrose.

An increased F:B ratio has been associated with obesity and increased energy harvest by the gut microbiota (Ley et al., 2005; Turnbaugh et al., 2006), although other studies have not replicated this in humans (Duncan et al., 2008; Hu et al., 2015). The increased F:B ratio in rLFD- and rHFD-fed mice relative to chow diet-fed mice in the present study suggests that the F:B ratio is altered by diet composition independently of obesity. Previous associations between increased F:B ratio and diet-induced obesity in mice are, potentially, a result of using chow diet as a control diet. We noted a large increase in the ileal F:B ratio in both the rLFD and rHFD groups. However, the members of the Firmicutes phyla in the ileum are distinct from those in the cecum.

Reduced microbiota diversity has been reported in humanized mice, colonized with human gut microbiota and fed a refined low-fiber diet compared to chow diet (Sonnenburg et al., 2016). Reductions in diversity were not observed in the present study. The use of mice with native microbiota may explain this if these are more resilient to surviving the effects of a low-fiber diet. The Shannon diversity index has been reported to be reduced in the cecum of mice fed an rHFD compared to chow diet (Turnbaugh et al., 2008). Our results did not show decreased microbial diversity in the ileum, cecum, colon, or fecal pellets, due to either diet or obesity indicating that, in this study, microbial diversity can be dissociated from the development of obesity.

A dominant *Bifidobacteria* (OTU8) increased only in the ileum and cecum of rLFD-fed mice (Figure 2C). *Bifidobacteria* have been reported to be reduced in obese mice (Cani et al., 2007). In the present study, three OTUs belonging to the *Erysipelotrichi* class, two belonging to the family *Erysipelotrichaceae* (OTU2 and OTU15), and a *Turicibacter* (OTU61) increased only in the rLFD-fed mice (Figure 3C). The *Erysipelotrichi* class has been reported to increase in diet-induced obese mice and obese human subjects (Turnbaugh et al., 2008). A *Deltaproteobacteria* (OTU6) was rare in chow diet-fed mice but highly represented in both rLFD- and rHFD-fed mice (Figures 2C and 3C). Proteobacteria have been proposed as a microbial signature of dysbiosis in the gut microbiota and reportedly increased in rHFD-fed mice (Shin et al., 2015). *Akkermansia muciniphila*, the sole member of the Verrucomicrobia phylum detected in our study, has previously been reported to be reduced in response to rHFD feeding (Everard et al., 2013). However, in the present study, its abundance was not altered by diet or obesity (Figure S1G). Only a small number of OTUs changed in proportional abundance between rHFD-fed and both chow diet- and rLFD-fed mice.

The large changes in microbiota composition between the chow diet- and rHFD-fed mice, and between the chow diet- and rLFD-fed mice, show the dramatic effect of altering the nutritional composition of the diet on the abundance of individual bacterial species. The lack of such large changes in microbiota composition between the rLFD and rHFD suggest that differences in fat and starch content between these diets was not as big a driver of bacterial microbiota composition as the absence of unrefined plant ingredients, compared to chow diet. These results indicate that the use of chow diet as a control diet to an rHFD can overestimate the changes in microbiota composition taking place in high-fat-diet-induced obesity in mice. Thus, changes in the proportional abundance of individual OTUs, as well as large compositional changes in gut microbiota, could be dissociated from rHFD-induced obesity in this study through the comparison with both the chow diet and rLFD control diets.

Increased energy harvest from dietary fiber in the form of cecal fermentation of SCFAs has been proposed to increase body fat in mice (Turnbaugh et al., 2006, 2008). Conventionalization of germ-free mice with a mouse microbiota also increases energy extraction from dietary fiber and body fat gain (Bäckhed et al., 2004). In contrast, increased SCFAs have been associated with lower food intake, reduced body weight, and improved metabolic health in mice when added to an rHFD (Everard et al., 2014; Van den Abbeele et al., 2011; Arora et al., 2012; den Besten et al., 2015). Cecal SCFAs were equally reduced in the present study in both rLFD-fed and rHFD-fed mice, compared to chow diet-fed mice. Furthermore, SCFA levels are significantly reduced in the cecum of obese rHFD-fed mice relative to lean chow diet-fed animals. These results question the concept of an increased microbial energy harvest of SCFAs as a contributor to diet-induced obesity, as the capacity of cecum and colonic energy extraction, observed through SCFA levels, is not different between obese and lean mice fed refined diets. Additionally, the reduction in SCFAs in the rLFD group without any increase in body fat suggests that, in the context of a diet low in fat, SCFAs are not essential to maintain body weight in mice. The reductions seen here in the length and weight of the cecum and colon confirms the similar reductions recently reported in mice fed a refined low-fiber diet (Chassaing et al., 2015). Additionally, we show here that the rLFD and rHFD also reduce the weight and length of the small intestine, a section of the gut not directly undergoing fiber fermentation.

These results disassociate alterations in gut microbiota composition, gut morphology, and short-chain fatty acids from

Figure 3. Effect of Chow Diet, rLFD, and rHFD on Cecum Microbiota Composition

(A) Heatmap of proportion of OTUs (≥3% abundance) in the cecum, with rows clustered by microbiota similarity using the Bray-Curtis calculator, and columns clustered by OTUs that occur more often together.

(B) OTU LDA values for chow diet-, rLFD-, and rHFD-fed mice (LDA score > 3) and heatmap showing relative abundance of each OTU between each mouse sample across rows; columns represent OTUs within each sample.

(C–E) Dot plots represent proportional abundance of the 5 OTUs with the highest LDA score in (C) the chow diet group, (D) the rLFD group, and (E) the rHFD group, with symbols each representing individual mice with mean ± SEM.

Significance was determined using Metastats in mothur. *$p < 0.05$; **$p < 0.01$; ***$p < 0.001$. n = 8 mice per group.

See also Figure S3 and Table S2.

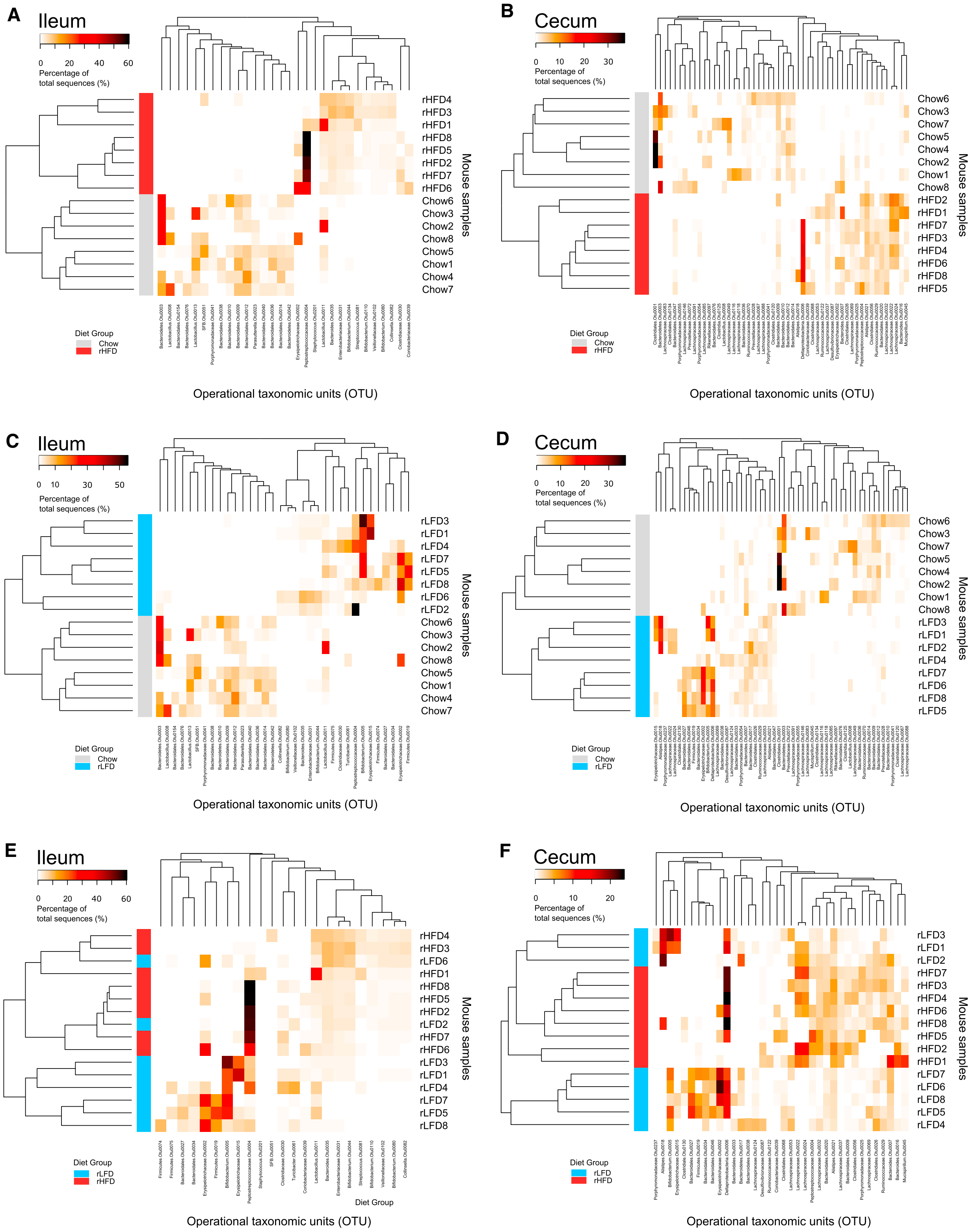

Figure 4. Effects of Individual Comparisons between Chow Diet, rLFD, and rHFD Groups

(A) Comparison of ileum microbiota between chow diet and rHFD.

(B) Comparison of cecal microbiota between chow diet and rHFD.

(legend continued on next page)

increases in body fat and glucose intolerance in mice fed an rHFD. The alterations in gut microbiota observed between chow diet and rHFD are likely due to the refined and semi-purified nutritional composition of the rHFD, contrasting with the unrefined chow diet. The broad changes in the microbiota composition between the chow diet- and rLFD-fed mice in the absence of body weight change lead us to question the links that have been drawn between changes in gut microbiota composition and obesity. In the context of similar protein and sucrose levels, substituting dietary fat for starch was the primary driver of body fat increase, while the lack of dietary fiber and overall nutritional composition shaped the gut microbiota composition, SCFA fermentation, and gut morphology independently from obesity. This study cannot show which of the many components present or absent in chow diet or refined diets were responsible for these changes; only that dietary fiber is likely to be an important contributor.

This study shows that the use of chow diet as the control diet, rather than a well-matched rLFD, can dramatically alter the results obtained, and the interpretation, of studies investigating the composition of the gut microbiota in rHFD-fed mice. This raises important questions about the prevailing understanding of the relationship between gut microbiota and obesity. These data also have broad implications for the interpretation of the many other rodent studies that involve the comparison of chow and refined high-fat diets, whether directly investigating the gut microbiota or where host physiology may be influenced by gut microbiota composition.

EXPERIMENTAL PROCEDURES

Ethical Approval and Institutional Governance

The experiments adhered to UK Home Office regulations according to the Animals (Scientific Procedures) Act, 1986, and were licensed by the UK Home Office under Project License PPL60/4359. The Project license was approved by the University of Aberdeen Animal Welfare and Ethical Review Body (Approval number ERC11-12:14), in accordance with the University Code of Practice for Research Involving the Use of Animals, and the scientific study was reviewed by the local Rowett Institute Scientific Management Committee (Approval number: 160715MD). The study was conducted at the Medical Research Facility of the University of Aberdeen, under the UK Home Office 2C (PEL) License number 60/2601. The mouse study was overseen by UK Home Office personal licencee I51A401DC (A.W.R.), with the availability of a permanent on-site veterinary surgeon.

Animals

Only male mice were used in this study. Forty-eight male C57BL/6J mice (JAX Mice Strain, Stock Number 000664) were purchased from Charles River Laboratories, UK, aged 7–8 weeks, and had been weaned and maintained on a VRF1 diet (Special Diet Services, Witham, Essex, UK). On arrival, mice were group housed in cages of 8–10 mice for a 1-week acclimatization period with sawdust bedding and a further week acclimatization to single housing; thus, mice were juvenile adults developmentally at the start of the dietary experiment. The room was maintained with a temperature of 21°C ± 2°C and a relative humidity of 55% ± 10%, and the lighting regime was a standard 12-hr:12-hr light:dark cycle, with lights on from 7 a.m. to 7 p.m. Food and water were provided *ad libitum*; food was provided as pelleted Rat and Mouse Breeder and Grower diet (Special Diet Services, Witham, Essex, UK).

Mouse Study Design

Mice were then individually housed for 1 week of acclimatization to individual housing, food intake measures and body weight measures. Mice were housed in shoebox cages with grid floors to allow spilt food to be collected on a tray below. Each cage contained a plastic tunnel, a roof-suspended house, and shredded paper for environmental enrichment. Three diets were used during the experiment: rodent chow (Chow), supplying energy as 9% fat, 22% protein, and 69% carbohydrate (Rat and Mouse Breeder and Grower, Special Diet Services, Witham, Essex, UK); rLFD (D12450J), supplying energy as 10% fat, 20% protein, and 70% carbohydrate; and rHFD (D12492), supplying energy as 60% fat, 20% protein, and 20% carbohydrate (Research Diets, New Brunswick, NJ, USA). During this acclimatization week, mice were fed the chow diet. Mice were randomized into one of the 3 diet groups: chow diet, rLFD, or rHFD. Mice were randomized using the Microsoft Excel "RAND" function. Briefly, a random number was generated for each mouse, the mice were ordered by the size of the random number (smallest to largest), and the mice were divided into three equal groups, being allocated for either chow diet, rLFD, or rHFD (n = 16 mice per group). Mice 1–8 in each diet group were preselected for gut microbiota analysis, and mice 9–16 in each diet group were preselected for intraperitoneal glucose tolerance tests. After the week of acclimatization to individual housing (9–10 weeks of age), mice were split into the 3 diet groups and offered the chow diet, rLFD, or rHFD *ad libitum* for 8 weeks.

Physiological Measurements

Body weight was recorded once per week. Body composition was determined once per week using EchoMRI (Echo Medical Systems, Houston, TX, USA), which provided total body fat and lean mass data. Food was weighed and added to the individual mice cages twice per week, when remaining food and spilled food were weighed. Weekly food intake was calculated accounting for spillage. Energy intake was calculated using the energy density of the diets, as kilojoules per gram, using published figures provided by manufacturers of the mouse diets.

Intraperitoneal Glucose Tolerance Test

An intraperitoneal glucose tolerance test (IPGTT) was carried out on 8 mice from each diet group after 8 weeks of experimental diet feeding. Mice were weighed and then fasted for 5 hr from 8:00 a.m. Glucose was prepared as 100 mg/mL in saline, filter sterilized, and stored at −20°C. An initial blood sample was taken at 0 min via a tail-tip cut removing 1 mm of tail tip prior to the intraperitoneal glucose injection containing 1.5 mg glucose per gram of body weight. Subsequently, blood samples were taken from the tail vein at 15, 30, 60, and 120 min by gently squeezing the tail to remove the scab and express a fresh spot of blood. The first spot of blood was discarded, and the second spot of blood was transferred directly from the tail onto an Accu-Chek Aviva Test Strip (Roche Diagnostics, Burgess Hill, UK). Blood glucose was measured using an Accu-Chek Aviva blood glucose monitor (Roche Diagnostics, UK). Areas under the curve for individual IPGTTs were calculated using SigmaPlot 13.0 (Systat Software, Hounslow, London, UK).

Intestinal Measurements and Sample Collection

Fecal pellets were collected at the end of the acclimatization week, when all mice were fed chow diet, and at the end of week 8. Briefly, fecal pellets were collected directly after the cages had been cleaned. Fresh fecal pellets

(C) Comparison of ileum microbiota between chow diet and rLFD.
(D) Comparison of cecal microbiota between chow diet and rLFD.
(E) Comparison of ileum microbiota between rLFD and rHFD.
(F) Comparison of cecal microbiota between rLFD and rHFD.
Heatmaps show the proportion of OTUs (≥3% abundance), with rows clustered by microbiota similarity using the Bray-Curtis calculator, and columns clustered by OTUs that occur more often together. n = 8 mice per group.
See also Figure S3 and Table S2.

OPEN ACCESS
CellPress

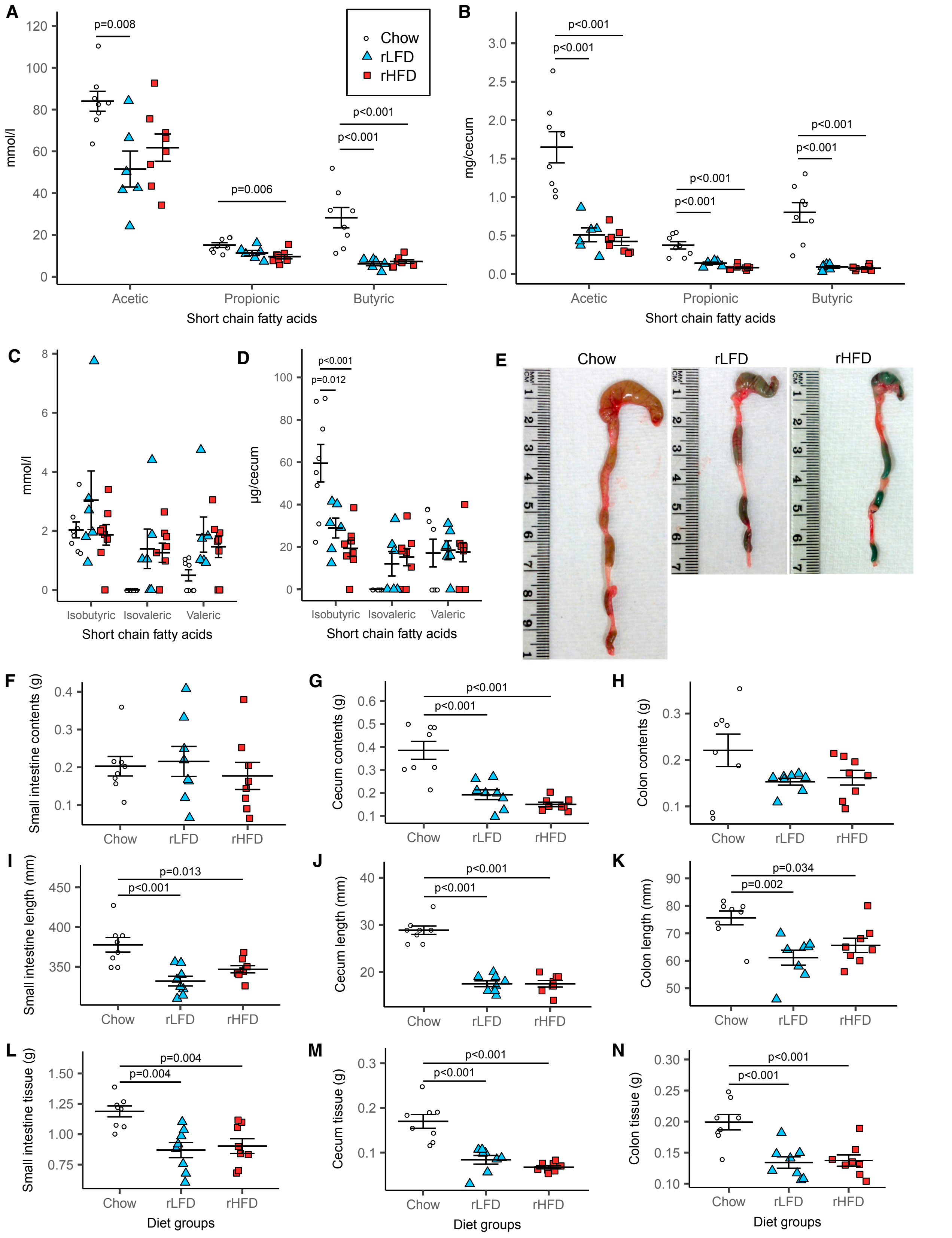

(legend on next page)

were collected as they fell through the cage grid floors until samples had been collected from all mice. Fecal pellets were placed on ice during the collection and then stored at −80°C. After 8 weeks of feeding experimental diets, mice, aged 17–18 weeks of age, were killed by terminal cardiac puncture under terminal anesthetic with isoflurane gas. The entire gastrointestinal tract was dissected, and fat and connective tissue were carefully removed. Tissue lengths were measured, full and empty weights were recorded, and photographs were taken. The luminal contents of the ileum, cecum, and colon were each collected, frozen on dry ice, and then stored at −80°C.

DNA Extraction

DNA was extracted from intestinal contents (terminal ileum, cecum, and colon), and fecal pellets. DNA was extracted using a FastDNA SPIN Kit for Feces (MP Biomedicals 116570200, MP Biomedicals SARL, Illkirch, France) and processed according to the manufacturer's instructions. Each sample DNA was eluted in 60 µL TED buffer. Eluted DNA concentration and purity were assessed using a NanoDrop ND-1000 spectrophotometer (Thermo Fisher Scientific, Wilmington, DE, USA) and agarose gel electrophoresis.

PCR Amplification for Sequencing

DNA extracted from mouse intestinal samples was used as a template for PCR amplification of the V1–V2 variable regions of the bacterial 16S rRNA gene using barcoded primers MiSeq-27F 5′-AATGATACGGCGACCACCGA GATCTACACTATGGTAATTCCAGGTTYGATYMTGGCTCAG-3′ and MiSeq-338R 5′-CAAGCAGAAGACGGCATACGAGAT-barcode-AGTCAGTCAGAAG CTGCCTCCCGTAGGAGT-3′ containing adaptors for downstream Illumina MiSeq sequencing. Each sample was PCR amplified with a reverse primer containing a unique (12-base) barcode and using New England BioLabs Q5 High-Fidelity DNA Polymerase. Each reaction mix contained 5× Q5 Reaction Buffer (5 µL), 10 mM dNTPs (0.5 µL), 10 µM 27F Primer (1.25 µL), 10 µM R Primer (1.25 µL), Q5 High-Fidelity DNA Polymerase (0.25 µL), DNA template (1 µL), and New England BioLabs sterile nucleotide water (15.75 µL) to a final volume of 25 µL. PCR reactions were set up in quadruplicate for each DNA sample. Thermocycling conditions for the PCR amplification were: 2 min at 98°C, then 20 cycles of 30 s at 98°C, 30 s at 50°C, and 90 s at 72°C; and, finally, a 5-min extension at 72°C, then a holding temperature of 4°C.

Following amplification, the quadruplicate PCR reactions were pooled into a single sterile 1.5-mL microcentrifuge tube. Pooled PCR samples were purified by ethanol precipitation. The DNA was resuspended in 30 µL TE buffer and stored at −20°C before quantification. The pooled PCR amplicons were quantified using a Qubit dsDNA HS Assay Kit (Invitrogen, CA, USA, Q32854). Briefly, a Working Solution was prepared equal to 199 µL Qubit HS buffer mixed with 1 µL Qubit HS reagent for each sample to be quantified. Two Qubit tubes were labeled HS1 and HS2, and 10 µL Qubit HS Standard 1 (0 ng/µL) and Qubit HS Standard 2 (10 ng/µL) were added to the tubes, respectively. 190 µL Working Solution was added to each tube, vortexed for 5 s, and incubated at room temperature for 2 min. The double-stranded DNA (dsDNA) HS setting was selected on the Qubit 2.0 Fluorometer (Invitrogen, CA, USA), and a new calibration using the two standard tubes was used to create a standard curve. Next 1 µL of sample was added into a Qubit Assay Tube (Invitrogen, CA, USA, Q32856), together with 199 µL Working Solution, vortexed for 5 s, and incubated at room temperature for 2 min, and then DNA concentration was determined using the Qubit 2.0 Fluorometer (Invitrogen, CA, USA). All sample concentrations were determined using the same Working Solution. After the 2-min incubation at room temperature, the concentration of each sample was measured in nanograms per microliter.

An equimolar mix was prepared for sequencing using equimolar concentrations of DNA from each sample. The amount of each sample to be added was calculated using the following formula: sample volume (in microliters) = DNA conc. of the sample with highest DNA conc. of all the samples (in nanograms per microliter)/DNA conc. of sample (in nanograms per microliter). All samples were above the minimum accepted concentration of 3 ng/µL. The Equimolar mix was then split into 2 equal volumes in 1.5-ml microcentrifuge tubes. One tube was stored at −20°C as a backup, while the other was prepared for sequencing.

The Equimolar mix was cleaned using gel purification to remove primer dimers using a Wizard SV Gel and PCR Clean-Up System (Promega, A9281, Madison, WI, USA). Briefly, the equimolar mix library was run out on a 1% agarose gel, stained with GelRed (Cambridge Bioscience, BT-41003, Munro House, Cambridge, UK), in Tris acetate-EDTA (TAE) buffer. Gel containing the band between ~300 and 400 bp was excised from the gel using a sterile scalpel and placed into pre-weighed 1.5-ml microcentrifuge tubes. The tubes were weighed to determine the weight of the gel slices. Into each tube was added 10 µL membrane binding solution per 10 mg of gel slice. The mix was vortexed to mix and incubated in a hot block at 65°C for 10 min or until the gel was fully dissolved. A maximum of 350 mg of sample in 350 µL binding solution was pipetted into the SV Minicolumn assembly (Filter Column plus collection tube) and left to bind for 1 min. This was then centrifuged for 1 min at 16,000 × *g* (Eppendorf 5415R, Hamburg, Germany). The liquid was discarded, then 700 µL Membrane Wash Solution was added to the column and then centrifuged for 1 min at 16,000 × *g* (Eppendorf 5415R, Hamburg, Germany). The flowthrough was discarded, and this was repeated with 500 µL Membrane Wash Solution and centrifuged for 6 min at 16,000 × *g* (Eppendorf 5415R, Hamburg, Germany), and the flowthrough was discarded. This was repeated until the DNA in all dissolved gel samples had been bound to the same column without eluting the DNA. The total DNA was eluted into a sterile 1.5-ml microcentrifuge tube by adding 50 µL Nuclease Free Water to the column, incubating at room temperature for 1 min, and then centrifuging for 1 min at 16,000 × *g*. The 50-µL volume was divided into two tubes, 25 µL in each; one stored at −20°C as a backup, while the other was used for sequencing.

Illumina MiSeq Sequencing

Paired-end sequencing of the pooled equimolar mix of PCR products was carried out on an Illumina MiSeq machine, using a read length of 2 × 250 bp. Illumina MiSeq sequencing was carried out by the Centre for Genome Enabled Biology and Medicine, University of Aberdeen.

Illumina MiSeq Sequence Data Analysis

The data obtained from Illumina MiSeq sequencing were analyzed using the mothur software package (Schloss et al., 2009) and based on the mothur MiSeq standard operating procedure (Kozich et al., 2013). A text file was created containing the paired reads, forward and reverse, generated by the Ilumina sequencing run for each sample. This included 3 DNA extraction kit blanks processed with only water as a control. The forward and reverse reads generated from the sequencing were assembled into paired contigs. After assembly, contigs were removed that were shorter than 280 base pairs or longer than 470 base pairs, contained ambiguous bases, or contained homopolymeric stretches of longer than 8 bases. One representative sequence for each unique sequence was extracted from the dataset. Unique sequences

Figure 5. Effects of Diet on Cecal Short-Chain Fatty Acids and Intestinal Morphology
(A) Cecal concentrations of acetic, propionic, and butyric acid.
(B) Total cecal acetic, propionic, and butyric acid.
(C) Cecal concentrations of isobutyric, isovaleric, and valeric acid.
(D) Total cecal isobutyric, isovaleric, and valeric acid.
(E) Cecum and colon morphology.
(F–H) Gut content weights in (F) the small intestine, (G) the cecum, and (H) the colon.
(I–K) The length of (I) the small intestine, (J) the cecum, and (K) the colon.
(L) Tissue weight of the small intestine, (M) the cecum, and (N) the colon.
Data indicate mean ± SEM. n = 8 mice per group; n = 6 mice in the rLFD group in (A)–(D).

were then grouped together and aligned against the SILVA reference database. OTUs were generated at a 97% similarity cutoff level. Due to a high number of unique singleton and doubleton sequences, representing 1 or 2 sequences in the dataset, these were removed to reduce the total number of sequences to less than 50,000, to reduce the file size to one that could be processed. Perseus (Quince et al., 2011) chimera removal software was used to detect and remove chimeric molecules created during PCR amplification. The Ribosomal Database Project (RDP) reference database (Release 10) (Wang et al., 2007) was used to assign taxonomic classifications to each OTU at the phylum, family, and genus levels. Spurious reads, including those derived from mitochondria, chloroplasts, or Eukarya, were removed from the dataset. Sequences classified as belonging to the *Lactococcus* genus were removed from the dataset. The final dataset contained a total of 2,694,538 sequences, with a range between samples of 4,495–39,081 sequences. Samples were then rarefied (sub-sampled) to reduce all 4,495 sequences to equalize the sequencing depth between all samples for later analysis. A representative sequence for each OTU was generated, and the most abundant OTUs were manually curated against the BLAST database.

Short-Chain Fatty Acid Analysis

The concentrations of short-chain fatty acids in cecal contents were determined by capillary gas chromatography. Frozen cecal contents were thawed on ice, and 100 mg of each sample was weighed out. Next, 200 μL distilled water was added to each 100 mg of sample and vortexed until suspended, before 75 μL 2-ethylbutyric acid (5 mmol/L) internal standard was added, and the sample was vortexed to mix. Samples were centrifuged at 16,000 × *g* for 20 min at 4°C (Eppendorf 5415R, Germany), and the supernatants were analyzed using an Agilent HP-FFAP column (Catalog No. 19095F-121) (dimensions: 10 m × 0.53 mm inner diameter [ID], 1 μm) gas chromatograph fitted with a fused silica capillary. Concentrations of acetate, propionate, butyrate, iso-butyrate, valerate, and iso-valerate were determined. The cecum contents samples from 2 mice in the rLFD groups were lost during processing and could not be used for short-chain fatty acid analysis.

Statistical Analysis

Unless otherwise stated, group comparisons for all data were performed using SigmaPlot 13.0 (Systat Software, Hounslow, London, UK). The normal distribution of sample data being analyzed was initially tested using the Shapiro-Wilk test. Sample groups that failed the Shapiro-Wilk test were plotted and visually assessed for normality. Comparisons between normally distributed samples were analyzed by one-way ANOVA followed by Tukey's post hoc test for multiple comparisons. Comparisons between skewed samples were analyzed using Kruskal-Wallis one-way ANOVA on ranks followed by Dunn's test for multiple comparisons. P values of 0.05 or less were considered significant. For microbiota sequence data, the LEfSe software tool (Segata et al., 2011), within the mothur software package (Schloss et al., 2009), was used to identify OTUs that differentiated the diet groups with a linear discriminant analysis (LDA) effect size of greater than 2. Metastats (White et al., 2009), a non-parametric t test, incorporating Fisher's exact test and the false discovery rate (FDR) was used to determine whether OTUs, or higher taxa, identified as having an LDA effect size of greater than 2, were significantly differentially represented between diet groups. P values generated using Metastats were corrected with the Benjamini-Hochberg method (Benjamini and Hochberg, 1995) to correct for the false discovery rate across multiple comparisons. P values < 0.05 were considered significant. The observed richness (Sobs), the estimated (Chao1) total richness, the Shannon diversity index, the inverse Simpson diversity index, and Good's coverage were used to calculate the bacterial diversity within each sample in the mothur software package (Schloss et al., 2009).

DATA AND SOFTWARE AVAILABILITY

The accession number for sequence data reported in this article is European Nucleotide Archive: ERP104030. The accession numbers for sequence data reported in this article are European Nucleotide Archive: ERS1906072–ERS1906191 (Table S2).

SUPPLEMENTAL INFORMATION

Supplemental Information includes four figures and two tables and can be found with this article online at https://doi.org/10.1016/j.celrep.2017.10.056.

AUTHOR CONTRIBUTIONS

Conceptualization, M.J.D., A.W.R., and P.J.M. Methodology, M.J.D., A.W.R., A.W.W., and P.J.M. Formal Analysis, M.J.D. Writing – Original Draft, M.J.D. Writing – Review & Editing, M.J.D., A.W.R., A.W.W., and P.J.M. Visualization, M.J.D. Supervision, M.J.D., A.W.R., A.W.W., and P.J.M. Funding Acquisition, A.W.R. and P.J.M.

ACKNOWLEDGMENTS

The authors gratefully acknowledge Doctoral Training Partnership funding from the BBSRC (M.J.D.) and funding from the Scottish Government (P.J.M., A.W.R., and A.W.W.). We also thank the Centre for Genome-Enabled Biology and Medicine for help with next-generation sequencing and Karen Garden and the Rowett's Analytical Services for SCFA analysis.

Received: May 25, 2017
Revised: September 5, 2017
Accepted: October 13, 2017
Published: November 7, 2017

REFERENCES

Arora, T., Loo, R.L., Anastasovska, J., Gibson, G.R., Tuohy, K.M., Sharma, R.K., Swann, J.R., Deaville, E.R., Sleeth, M.L., Thomas, E.L., et al. (2012). Differential effects of two fermentable carbohydrates on central appetite regulation and body composition. PLoS ONE *7*, e43263.

Bäckhed, F., Ding, H., Wang, T., Hooper, L.V., Koh, G.Y., Nagy, A., Semenkovich, C.F., and Gordon, J.I. (2004). The gut microbiota as an environmental factor that regulates fat storage. Proc. Natl. Acad. Sci. USA *101*, 15718–15723.

Benjamini, Y., and Hochberg, Y. (1995). Controlling the false discovery rate: a practical and powerful approach to multiple testing. J.R. Stat. Soc. Series B *57*, 289–300.

Benoit, B., Plaisancié, P., Awada, M., Géloën, A., Estienne, M., Capel, F., Malpuech-Brugère, C., Debard, C., Pesenti, S., Morio, B., et al. (2013). High-fat diet action on adiposity, inflammation, and insulin sensitivity depends on the control low-fat diet. Nutr. Res. *33*, 952–960.

Black, B.L., Croom, J., Eisen, E.J., Petro, A.E., Edwards, C.L., and Surwit, R.S. (1998). Differential effects of fat and sucrose on body composition in A/J and C57BL/6 mice. Metabolism *47*, 1354–1359.

Buettner, R., Schölmerich, J., and Bollheimer, L.C. (2007). High-fat diets: modeling the metabolic disorders of human obesity in rodents. Obesity (Silver Spring) *15*, 798–808.

Byrne, C.S., Chambers, E.S., Morrison, D.J., and Frost, G. (2015). The role of short chain fatty acids in appetite regulation and energy homeostasis. Int. J. Obes. *39*, 1331–1338.

Cani, P.D., Amar, J., Iglesias, M.A., Poggi, M., Knauf, C., Bastelica, D., Neyrinck, A.M., Fava, F., Tuohy, K.M., Chabo, C., et al. (2007). Metabolic endotoxemia initiates obesity and insulin resistance. Diabetes *56*, 1761–1772.

Chassaing, B., Miles-Brown, J., Pellizzon, M., Ulman, E., Ricci, M., Zhang, L., Patterson, A.D., Vijay-Kumar, M., and Gewirtz, A.T. (2015). Lack of soluble fiber drives diet-induced adiposity in mice. Am. J. Physiol. Gastrointest. Liver Physiol. *309*, G528–G541.

David, L.A., Maurice, C.F., Carmody, R.N., Gootenberg, D.B., Button, J.E., Wolfe, B.E., Ling, A.V., Devlin, A.S., Varma, Y., Fischbach, M.A., et al. (2014). Diet rapidly and reproducibly alters the human gut microbiome. Nature *505*, 559–563.

den Besten, G., Bleeker, A., Gerding, A., van Eunen, K., Havinga, R., van Dijk, T.H., Oosterveer, M.H., Jonker, J.W., Groen, A.K., Reijngoud, D.J., and Bakker, B.M. (2015). Short-chain fatty acids protect against high-fat diet-induced

obesity via a PPARγ-dependent switch from lipogenesis to fat oxidation. Diabetes *64*, 2398–2408.

Dollive, S., Chen, Y.Y., Grunberg, S., Bittinger, K., Hoffmann, C., Vandivier, L., Cuff, C., Lewis, J.D., Wu, G.D., and Bushman, F.D. (2013). Fungi of the murine gut: episodic variation and proliferation during antibiotic treatment. PLoS ONE *8*, e71806.

Duncan, S.H., Lobley, G.E., Holtrop, G., Ince, J., Johnstone, A.M., Louis, P., and Flint, H.J. (2008). Human colonic microbiota associated with diet, obesity and weight loss. Int. J. Obes. *32*, 1720–1724.

Everard, A., Belzer, C., Geurts, L., Ouwerkerk, J.P., Druart, C., Bindels, L.B., Guiot, Y., Derrien, M., Muccioli, G.G., Delzenne, N.M., et al. (2013). Cross-talk between *Akkermansia muciniphila* and intestinal epithelium controls diet-induced obesity. Proc. Natl. Acad. Sci. USA *110*, 9066–9071.

Everard, A., Lazarevic, V., Gaïa, N., Johansson, M., Ståhlman, M., Backhed, F., Delzenne, N.M., Schrenzel, J., François, P., and Cani, P.D. (2014). Microbiome of prebiotic-treated mice reveals novel targets involved in host response during obesity. ISME J. *8*, 2116–2130.

Hu, H.-J., Park, S.-G., Jang, H.B., Choi, M.-K., Park, K.-H., Kang, J.H., Park, S.I., Lee, H.J., and Cho, S.H. (2015). Obesity alters the microbial community profile in Korean adolescents. PLoS One *10*, e0134333.

Kozich, J.J., Westcott, S.L., Baxter, N.T., Highlander, S.K., and Schloss, P.D. (2013). Development of a dual-index sequencing strategy and curation pipeline for analyzing amplicon sequence data on the MiSeq Illumina sequencing platform. Appl. Environ. Microbiol. *79*, 5112–5120.

Ley, R.E., Bäckhed, F., Turnbaugh, P., Lozupone, C.A., Knight, R.D., and Gordon, J.I. (2005). Obesity alters gut microbial ecology. Proc. Natl. Acad. Sci. USA *102*, 11070–11075.

Ley, R.E., Turnbaugh, P.J., Klein, S., and Gordon, J.I. (2006). Microbial ecology: human gut microbes associated with obesity. Nature *444*, 1022–1023.

Quince, C., Lanzen, A., Davenport, R.J., and Turnbaugh, P.J. (2011). Removing noise from pyrosequenced amplicons. BMC Bioinformatics *12*, 38.

Ricci, M. (2013). The Importance of a Proper Control Diet (Res. Diets Focus).

Ridaura, V.K., Faith, J.J., Rey, F.E., Cheng, J., Duncan, A.E., Kau, A.L., Griffin, N.W., Lombard, V., Henrissat, B., Bain, J.R., et al. (2013). Gut microbiota from twins discordant for obesity modulate metabolism in mice. Science *341*, 1241214.

Schloss, P.D., Westcott, S.L., Ryabin, T., Hall, J.R., Hartmann, M., Hollister, E.B., Lesniewski, R.A., Oakley, B.B., Parks, D.H., Robinson, C.J., et al. (2009). Introducing mothur: open-source, platform-independent, community-supported software for describing and comparing microbial communities. Appl. Environ. Microbiol. *75*, 7537–7541.

Segata, N., Izard, J., Waldron, L., Gevers, D., Miropolsky, L., Garrett, W.S.W., Huttenhower, C., Golub, T., Petricoin, E., Ardekani, A., et al. (2011). Metagenomic biomarker discovery and explanation. Genome Biol. *12*, R60.

Shin, N.R., Whon, T.W., and Bae, J.W. (2015). Proteobacteria: microbial signature of dysbiosis in gut microbiota. Trends Biotechnol. *33*, 496–503.

Sonnenburg, E.D., Smits, S.A., Tikhonov, M., Higginbottom, S.K., Wingreen, N.S., and Sonnenburg, J.L. (2016). Diet-induced extinctions in the gut microbiota compound over generations. Nature *529*, 212–215.

Stenman, L.K., Holma, R., and Korpela, R. (2012). High-fat-induced intestinal permeability dysfunction associated with altered fecal bile acids. World J. Gastroenterol. *18*, 923–929.

Turnbaugh, P.J., Ley, R.E., Mahowald, M.A., Magrini, V., Mardis, E.R., and Gordon, J.I. (2006). An obesity-associated gut microbiome with increased capacity for energy harvest. Nature *444*, 1027–1031.

Turnbaugh, P.J., Bäckhed, F., Fulton, L., and Gordon, J.I. (2008). Diet-induced obesity is linked to marked but reversible alterations in the mouse distal gut microbiome. Cell Host Microbe *3*, 213–223.

Turnbaugh, P.J., Ridaura, V.K., Faith, J.J., Rey, F.E., Knight, R., and Gordon, J.I. (2009). The effect of diet on the human gut microbiome: a metagenomic analysis in humanized gnotobiotic mice. Sci. Transl. Med. *1*, 6ra14.

Van den Abbeele, P., Gérard, P., Rabot, S., Bruneau, A., El Aidy, S., Derrien, M., Kleerebezem, M., Zoetendal, E.G., Smidt, H., Verstraete, W., et al. (2011). Arabinoxylans and inulin differentially modulate the mucosal and luminal gut microbiota and mucin-degradation in humanized rats. Environ. Microbiol. *13*, 2667–2680.

Wang, Q., Garrity, G.M., Tiedje, J.M., and Cole, J.R. (2007). Naive Bayesian classifier for rapid assignment of rRNA sequences into the new bacterial taxonomy. Appl. Environ. Microbiol. *73*, 5261–5267.

Warden, C.H., and Fisler, J.S. (2008). Comparisons of diets used in animal models of high-fat feeding. Cell Metab. *7*, 277.

West, D.B., Waguespack, J., and McCollister, S. (1995). Dietary obesity in the mouse: interaction of strain with diet composition. Am. J. Physiol. *268*, R658–R665.

White, J.R., Nagarajan, N., and Pop, M. (2009). Statistical methods for detecting differentially abundant features in clinical metagenomic samples. PLoS Comput. Biol. *5*, e1000352.

Cell Reports, Volume *21*

Supplemental Information

Dietary Uncoupling of Gut Microbiota and Energy Harvesting from Obesity and Glucose Tolerance in Mice

Matthew J. Dalby, Alexander W. Ross, Alan W. Walker, and Peter J. Morgan

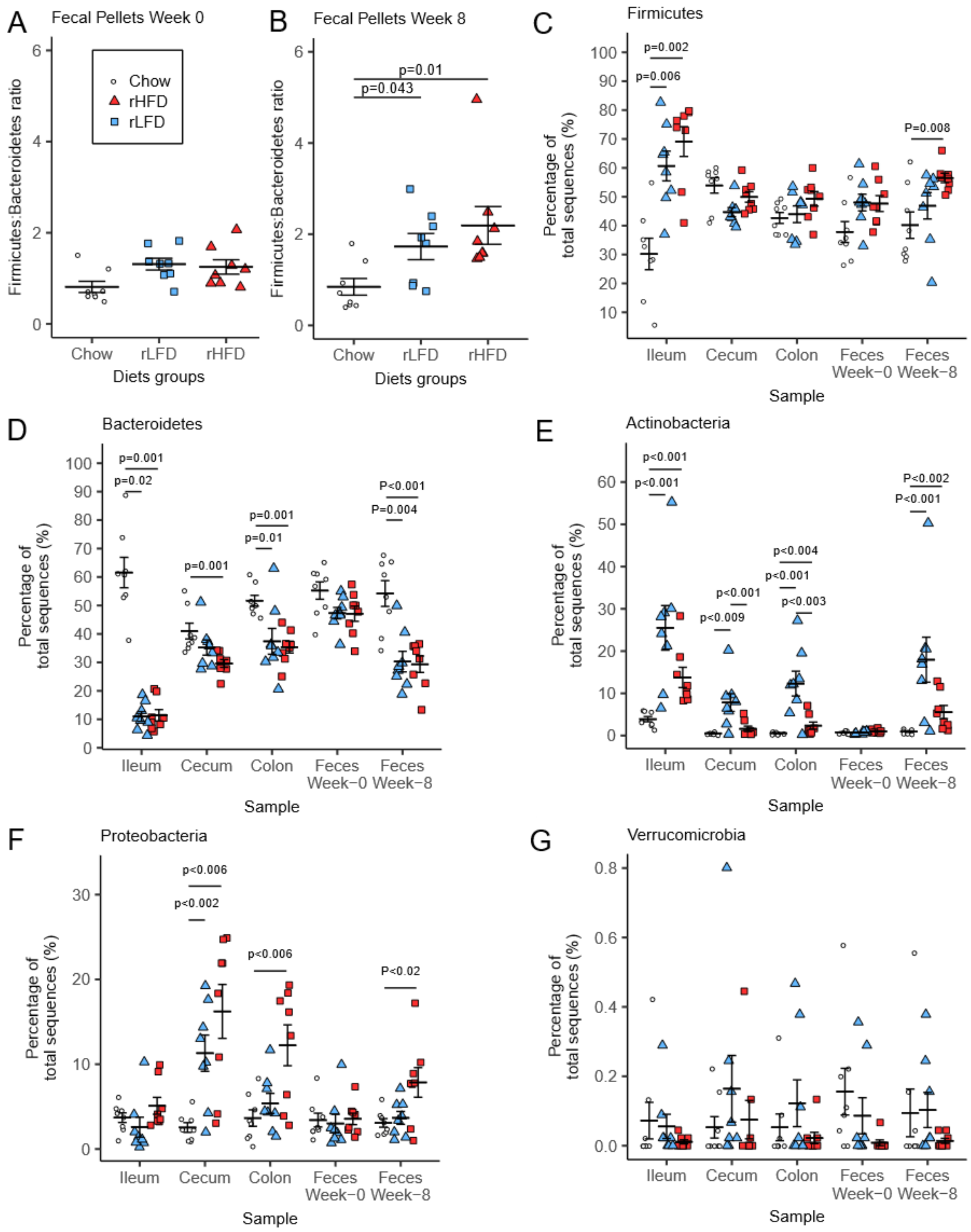

Figure S1. Initial F:B ratio and phylum proportional abundance. Related to Figure 1.

(A) Firmicutes:Bacteroidetes (F:B) ratio at Week 0. (B) F:B ratio at Week 8. Proportional abundance of the (C) Firmicutes phylum, (D) the Bacteroidetes phylum, (E) the Actinobacteria phylum, (F) the Proteobacteria phylum, and (G) the Verrucomicrobia phylum. Data represent mean ± SEM. n = 8 mice/group.

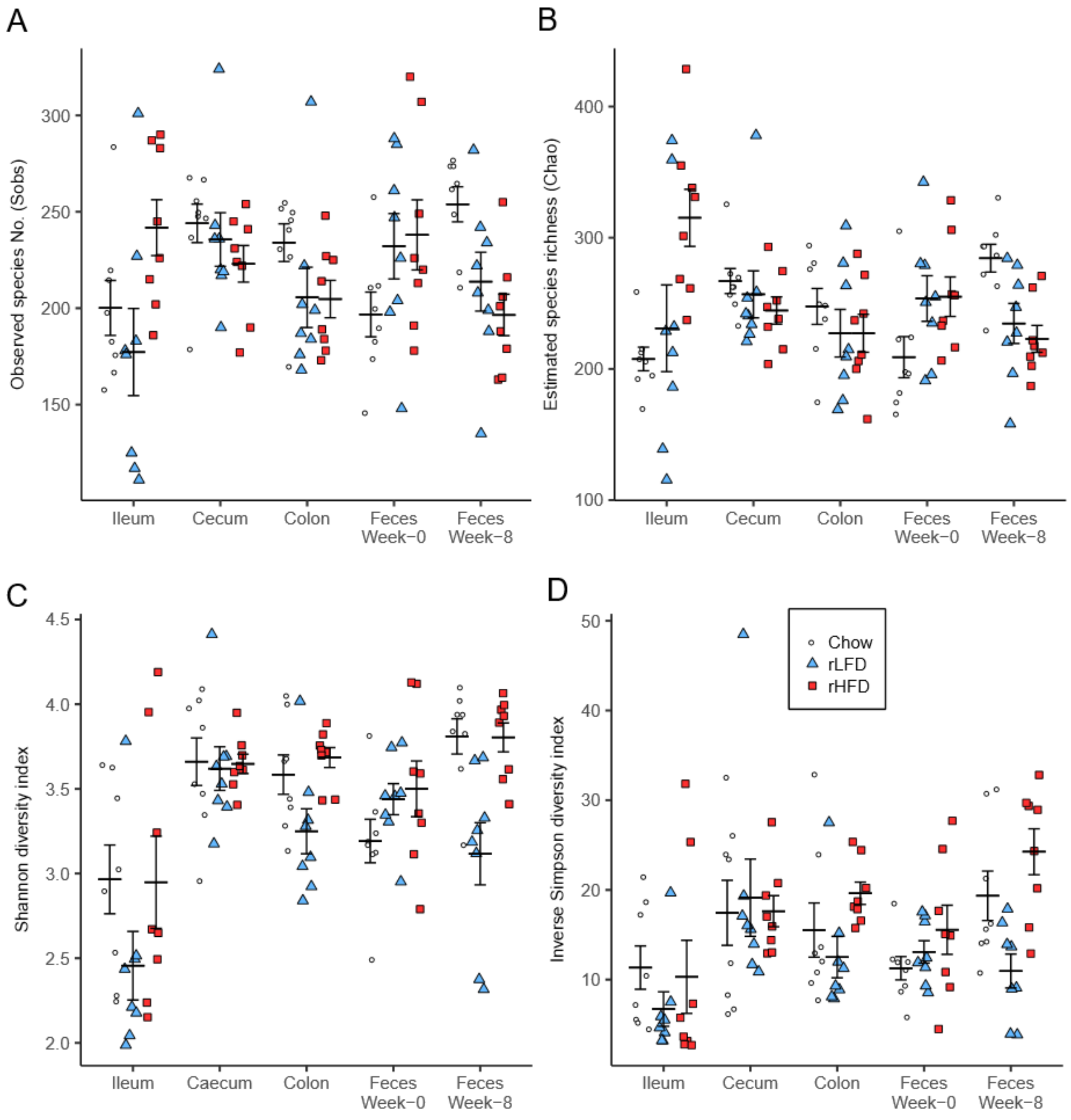

Figure S2. Measures of microbiota diversity. Related to Figure 1.

(A) Observed species richness (Sobs). (B) Estimated total species richness (Chao). (C) Shannon diversity index. (D) Inverse Simpson diversity index. Data represent mean ± SEM. n = 8 mice/group.

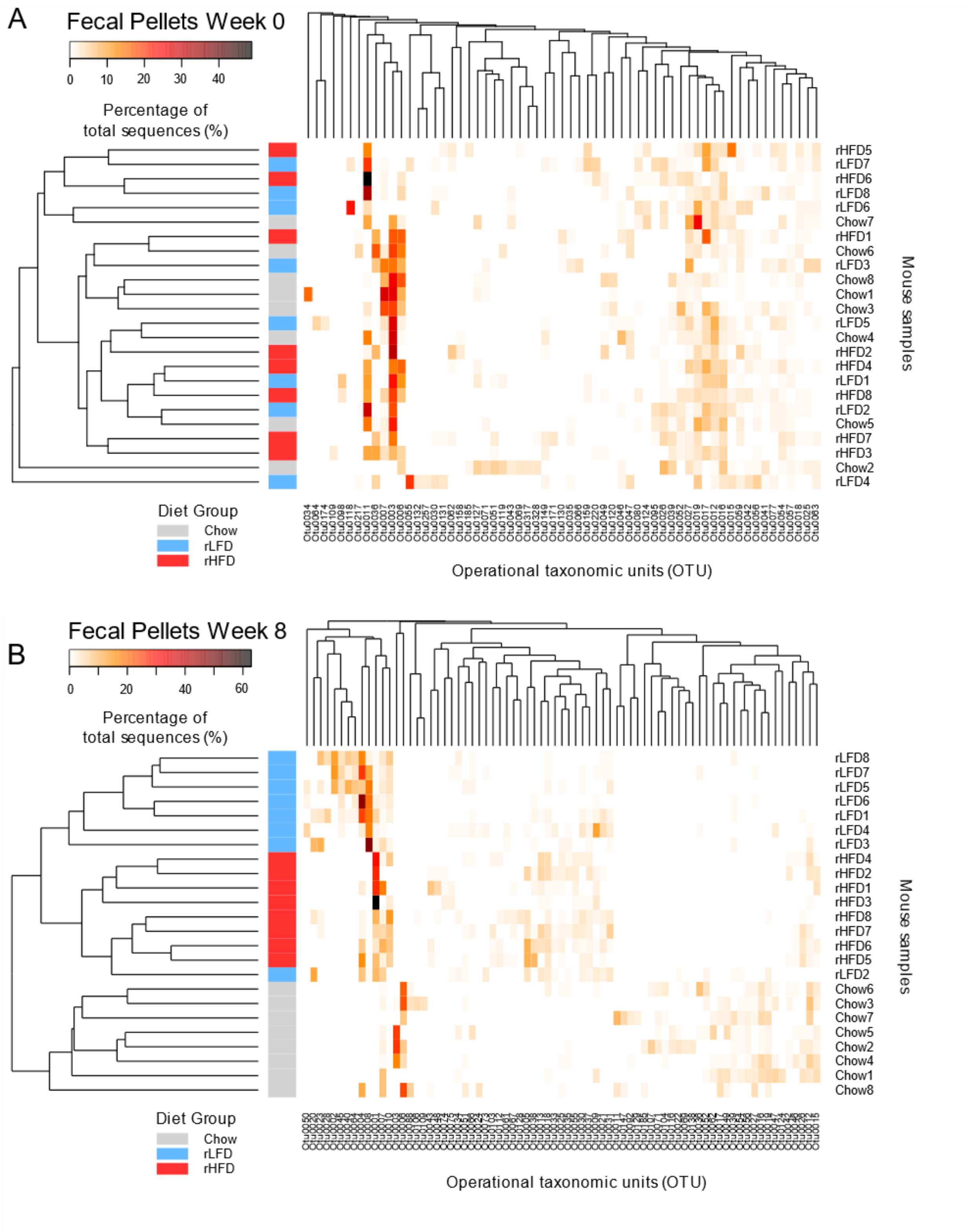

Figure S3. Effect of diet on fecal pellet microbiota composition. Related to Figure 3.

(A) Fecal pellet microbiota composition at Week 0. (B) Fecal pellet microbiota composition at Week 8. Heat maps show proportions of OTUs (>3% abundance) in the fecal pellets, with rows clustered by microbiota similarity using the Bray-Curtis calculator, and columns clustered by OTUs that occur more often together in a sample. n = 8 mice/group.

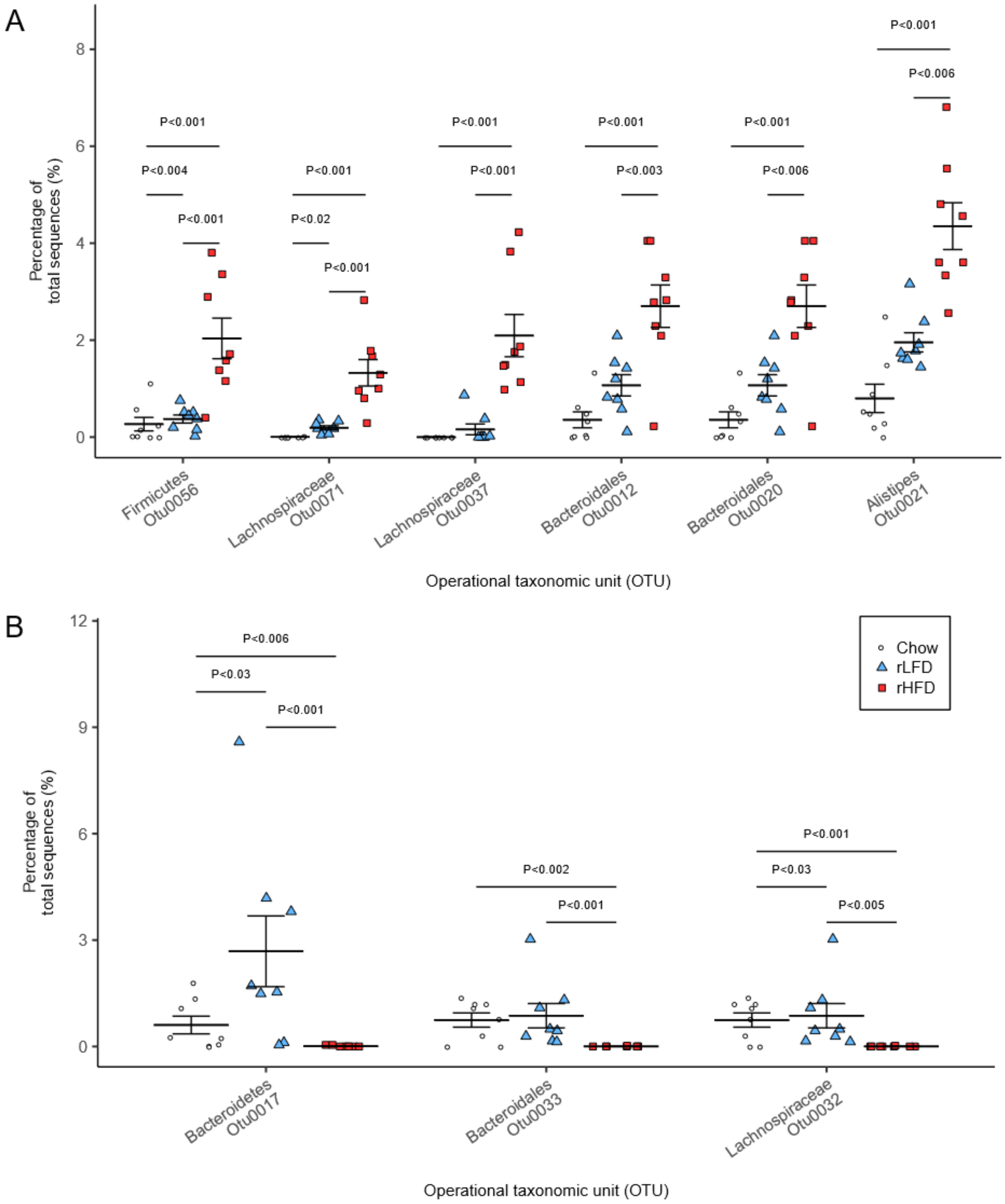

Figure S4. Cecal OTUs with abundance significantly altered only in rHFD fed mice. Related to Figure 4.

(A) OTUs (>0.5% proportional abundance) with significantly higher proportional abundance in rHFD fed mice compared to both Chow and rLFD fed mice. (B) OTUs (>0.5% proportional abundance) with significantly lower proportional abundance in rHFD fed mice compared to both Chow and rLFD fed mice. Statistical significance determined using Metastats in the mothur program. Data represent mean ± SEM. n = 8 mice/group.
